# Systematic review of pituitary gland and pituitary adenoma automatic segmentation techniques in magnetic resonance imaging

**DOI:** 10.3389/fradi.2026.1737075

**Published:** 2026-04-10

**Authors:** Mubaraq Yakubu, Navodini Wijithilake, Jonathan Shapey, Andrew King, Alexander Hammers

**Affiliations:** 1 King’s College London & Guy’s and St Thomas’ PET Centre, London, United Kingdom; 2 Radiology Department, Aminu Kano Teaching Hospital, Kano, Nigeria; 3 Medical Artificial Intelligence (MAI) Lab, Crestview Radiology-NOHIL CT and MRI Centre, Lagos, Nigeria; 4 Biomedical Engineering and Imaging Science, King’s College London, London, United Kingdom; 5 Research Department of Biomedical Computing, School of Biomedical Engineering and Imaging Sciences, King’s College London, London, United Kingdom; 6 Research Department of Early Life Imaging, School of Biomedical Engineering and Imaging Sciences, King’s College London, London, United Kingdom

**Keywords:** automatic segmentation, deep learning, magnetic resonance imaging, pituitary adenoma, pituitary gland, semi-automatic segmentation, U-Net

## Abstract

Accurate segmentation of both the pituitary gland and adenomas from magnetic resonance imaging (MRI) is essential for diagnosis and treatment of pituitary adenomas. This systematic review evaluates automatic segmentation methods for improving the accuracy and efficiency of MRI-based segmentation of pituitary adenomas and the gland itself. We analysed 34 studies that employed automatic and semi-automatic segmentation methods out of 353 reviewed studies. We extracted and synthesized data on segmentation techniques and performance metrics (such as Dice overlap scores). The majority of reviewed studies utilized deep learning approaches, with U-Net-based models being the most prevalent. Automatic methods yielded Dice scores of 0%–89% for pituitary gland and 4%–96% for adenoma segmentation. Semi-automatic methods reported 80%–92% for pituitary gland and 75%–88% for adenoma segmentation. Most studies did not report important metrics such as MR field strength, age and adenoma size (macro/micro/giant) or even adenoma type and human subject numbers. Automated segmentation techniques such as U-Net-based models show promise, especially for adenoma segmentation, but further improvements are needed to achieve consistently good performance in small structures like the normal pituitary gland. Future progress will require methodological innovation and larger, more diverse datasets to enhance clinical applicability.

**Systematic Review Registration:** https://www.crd.york.ac.uk/PROSPERO/view/CRD42023407127, PROSPERO CRD42023407127.

## 1 Introduction

Pituitary adenomas (PAs), also known as pituitary neuroendocrine tumours (1), are heterogeneous, slow-growing brain tumours arising from abnormal growth of the pituitary gland (PG). PAs are relatively common, occurring in approximately 14% of autopsy studies (2), but almost all are benign (3). Functional PAs, which are often classified based on size as microadenomas (<1 cm), may cause debilitating hormonal disorders such as Cushing’s disease (4). In contrast, larger non-functional adenomas classified as macroadenomas (>1 cm) and giant PAs (>4 cm) can exert mass effects, leading to headaches and visual disturbances by compressing adjacent structures including the optic chiasm and cavernous sinus (5–8).

Magnetic resonance imaging (MRI) is the modality of choice for the clinical investigation of PAs (9). Common MRI sequences include T1-weighted, T2-weighted, and Fluid Attenuated Inverted Recovery (FLAIR) (10). Contrast medium such as gadolinium-DTPA, typically at 0.1 mmol/kg, enhances delineation of PAs and surrounding structures, especially in small functional PAs (11, 12). T1- and T2-weighted imaging generally provide satisfactory visualization (13), but variability in acquisition parameters and sequence choice can affect diagnostic quality (14). Despite these imaging nuances, there is limited availability of curated public datasets for algorithm development when compared to other brain tumours (15), with the *Cheng 2015 dataset*, also known as Figshare dataset (16, 17), being a rare exception.

In the surgical context, accurate segmentation of PG and PA provides critical preoperative information, including volumetric assessment of tumour burden, spatial relationships to adjacent structures (e.g., optic chiasm and cavernous sinus), and delineation of residual healthy pituitary tissue, all of which directly inform surgical approach selection, extent of resection, and risk stratification for postoperative endocrine dysfunction (18, 19). Although PG and PA are anatomically contiguous, they present distinct segmentation challenges and clinical objectives (20). PG segmentation targets a small, anatomically variable structure with low tissue contrast, where precise boundary delineation is essential for radiotherapy planning, endocrine assessment, and longitudinal volumetric analysis (21). In contrast, PA segmentation focuses on lesion delineation, where tumour size, extent, and invasion of adjacent structures directly inform surgical planning, risk stratification, and treatment response evaluation (22). Despite these differences, PG and PA segmentation are often addressed within the same imaging examinations and surgical workflows, where accurate delineation of both the residual healthy gland and adenoma is required (23). Traditional approaches such as thresholding, clustering, atlasing, and model-based methods laid the foundation for automated segmentation (24), but the advent of deep learning has transformed the field (25). Architectures such as U-Net (26) and its derivatives now dominate medical image segmentation and have shown strong performance in brain tumour applications (27). Their application to PG and PA imaging holds promise for improved precision, reproducibility, and efficiency in neuroendocrine and neurosurgical workflows (28).

There is considerable variation across studies investigating automatic segmentation of the PG and PA in MRI, spanning methodological approaches, model architectures, imaging protocols, dataset composition, evaluation metrics, and reporting of key clinical and technical parameters, which complicates direct comparison and assessment of clinical readiness across segmentation approaches. To our knowledge, despite their central importance in neuroendocrine and neurosurgical care, this is the first systematic review to comprehensively evaluate both automated and semi-automated segmentation methods for these structure. While previous reviews have examined broader applications of deep learning in neuro-oncology (29–32), none have provided a focused synthesis of segmentation approaches specific to PG and PA. The aim of this review is therefore to systematically evaluate the literature on PG and PA segmentation using MRI, highlight the strengths and limitations of existing methods, and identify priorities for improving their reliability and readiness for clinical implementation. In this review, key methodological strengths and limitations identified across studies are summarized thematically within the Discussion. Accordingly, this work was designed as a systematic review with narrative synthesis, conducted in accordance with PRISMA guidelines. Owing to substantial heterogeneity in reported outcome units (e.g., subjects, images, slices, volumes), overlapping patient cohorts, and inconsistent reporting of variance measures, a formal meta-analysis with pooled effect estimates was neither feasible nor appropriate. Instead, the review focuses on qualitative comparison and structured descriptive synthesis of reported segmentation performance across studies.

## 2 Methods

### 2.1 Study design

This systematic review was conducted in accordance with PRISMA guidelines (33) and registered in PROSPERO (ID CRD42023407127). The research question was structured using a PICOS framework (34):—**Population:** patients with pituitary adenomas or normal pituitary glands imaged by MRI.—**Intervention:** automated or semi-automated segmentation methods.—**Comparator:** manual delineation or alternative computational approaches.—**Outcomes:** segmentation performance metrics, particularly the Dice similarity coefficient.—**Study design:** original research articles reporting quantitative results using clinical or publicly available datasets.

### 2.2 Eligibility criteria

We included full-length peer-reviewed articles focused on segmentation of PG or PA using MRI. Eligible studies described an automatic, semi-automatic, or deep learning algorithm targeting the pituitary or sellar region. Studies were excluded if they were case reports, reviews, conference abstracts, book chapters, meta-analyses, or editorials, or if they did not report the use of manual or automatic segmentation or MRI acquisition protocols in patients with PG or PA imaging. Peer-reviewed conference proceedings published as full-length articles with complete methodological descriptions and quantitative results were considered eligible, whereas short abstracts or extended summaries were excluded.

### 2.3 Information sources

The literature search was performed in three major databases: PubMed, Web of Science, and Scopus. Reference lists of included studies were also screened. There were no restrictions on the search; however, the last search update was conducted on October 21, 2024.

### 2.4 Search strategy

The search combined controlled vocabulary and free-text terms related to the pituitary gland and adenomas, MRI, and segmentation methods. The search string was:

(‘‘Pituitary’’ OR ‘‘Pituitary Adenoma*’’ OR ‘‘Pituitary Microadenoma’’ OR ‘‘Pituitary Macroadenoma’’ OR ‘‘Pituitary Tumo*’’ OR ‘‘Hypophysis’’ OR ‘‘Hypophysis Cerebri’’ OR ‘‘Hypophysis Tumo*’’ OR ‘‘Hypophysis Cerebri Tumo*’’ OR ‘‘Adenoma*’’) AND (‘‘Magnetic Resonance Imaging’’ OR ‘‘MRI’’ OR ‘‘MR’’) AND (‘‘Segmentation’’ OR ‘‘Manual Segmentation’’ OR ‘‘Automatic Segmentation’’ OR ‘‘Semi-Automatic Segmentation’’).

### 2.5 Selection process

Two independent reviewers screened titles and abstracts using the Rayyan web application (35). Full texts were retrieved for studies meeting eligibility criteria. Disagreements were resolved through discussion with a third reviewer. Studies were included if they: (1) aimed to delineate PG, PA, or the sellar region, (2) used MRI with automatic or semi-automatic segmentation tools, and (3) reported segmentation performance metrics.

### 2.6 Data collection process

Data extraction was performed independently by two reviewers using a pre-defined spreadsheet. Extracted variables included study design, population characteristics, imaging sequences, segmentation method, reported performance metrics, and the number of human subjects when explicitly reported. When subject counts were not provided, or could not be disaggregated at the pituitary-specific level (e.g., public multi-class datasets), this was recorded as not reported (NR). Discrepancies were resolved in discussion with a third reviewer. For papers that reported both objective metrics (e.g., Dice coefficient, Hausdorff distance) and subjective evaluations (e.g., expert assessments), only the objective measurements were collected.

### 2.7 Data items

The main outcomes were segmentation performance metrics, including: Dice score, Jaccard index, Matthews correlation coefficient, kappa score, area under the curve (AUC), relative volume error, average Hausdorff distance (AHD), average symmetric surface distance (ASSD), precision, accuracy, sensitivity, and specificity. Not all studies reported every metric, the Dice score was the most consistently available and was therefore the primary measure used for analysis. The Dice similarity coefficient quantifies the spatial overlap between automated segmentation outputs (predictions) and reference (typically expert manual ground truth) segmentations, with values closer to 1 indicating greater agreement.

### 2.8 Public datasets used in included studies

Several of the included studies utilized publicly available MRI datasets for pituitary gland or adenoma segmentation. Among these, the most widely used was the *Cheng dataset*, which served as a benchmark for many deep learning models. Other public datasets were limited in size or accessibility, with most studies instead relying on single-institution or private clinical data.

#### 2.8.1 Cheng dataset description

Several studies in this review employed the *Cheng 2015 dataset* (16), which provides T1-weighted contrast-enhanced MRI scans of the pituitary region. The full dataset consists of 3,064 2D slices from 233 patients and includes three types of brain tumors: 930 pituitary, 708 meningioma, and 1,426 glioma slices. The images were acquired between September 2,005 and October 2010 at Nanfang Hospital (Guangzhou, China) and the General Hospital of Tianjin Medical University (Tianjin, China). Each scan has an in-plane resolution of 512 × 512 pixels, with a pixel spacing of 0.49 × 0.49 mm^2^, a slice thickness of 6 mm, and an inter-slice gap of 1 mm. A gadolinium-based contrast agent (Gd-DTPA) was administered at a standard dose of 0.1 mmol/kg at an injection rate of 2 mL/s.

Only segmentation performance metrics explicitly reported for pituitary adenoma cases were extracted from studies using the Cheng dataset; results derived from other tumour classes (e.g., meningioma or glioma) were not included in the analysis.

### 2.9 Risk of bias assessment

The risk of bias in included studies was evaluated using the Mixed Methods Appraisal Tool (MMAT) (36). Criteria assessed included appropriateness of study design, sampling, data collection, data analysis, and researcher influence. Scores were summarized as low risk (>70%), moderate risk (50%–70%), or high risk (<50%).

### 2.10 Certainty of evidence

Certainty of evidence was assessed based on risk of bias, reproducibility of results across cohorts, relevance to the research question, study design quality, and completeness of reporting. Evidence was graded as high, moderate, low, or very low certainty (refer to Supplementary Material for detailed information).

### 2.11 Data synthesis

We planned to synthesize data using quantitative meta-analysis only if studies reported comparable outcome measures, variance estimates, and consistent units of analysis. However, due to substantial heterogeneity in study designs, imaging protocols, outcome definitions, and reporting units, this approach was not feasible. Accordingly, no quantitative meta-analysis (i.e., pooled effect estimation using fixed- or random-effects models) was performed.

Instead, results were synthesized using a narrative, study-level descriptive approach, summarizing reported segmentation performance metrics and methodological characteristics across studies. No pooled Dice coefficient, summary effect size, or heterogeneity statistic was calculated.

### 2.12 Statistical analysis

Statistical analyses were conducted solely as exploratory, unweighted comparisons at the study level to aid interpretation of reported performance trends and to highlight current reporting limitations in the literature. A weighted meta-analytic approach was not applied because sample sizes and units of analysis (patients, images, slices, or volumes) were inconsistently reported, and several studies evaluated multiple models on overlapping cohorts.

The unit of analysis was the reported summary Dice coefficient per study, rather than individual subjects or images. These analyses were not intended to estimate pooled performance, infer population-level effects, or substitute for a formal meta-analysis.

To evaluate potential differences in reported segmentation performance between study groups, non-parametric statistical methods were used. Owing to variability in sample sizes and the non-normal distribution of reported metrics, the Mann–Whitney U test compared median Dice similarity coefficients between automatic and semi-automatic methods separately for PG and PA segmentation. The Kolmogorov–Smirnov test assessed differences in the overall distribution of reported performance scores. All tests were two-tailed, with a significance threshold set at p<0.05. All statistical results were therefore interpreted descriptively rather than as inferential meta-analytic findings.

## 3 Results

### 3.1 Study selection

#### 3.1.1 Search and selection process

**Figure 1:**
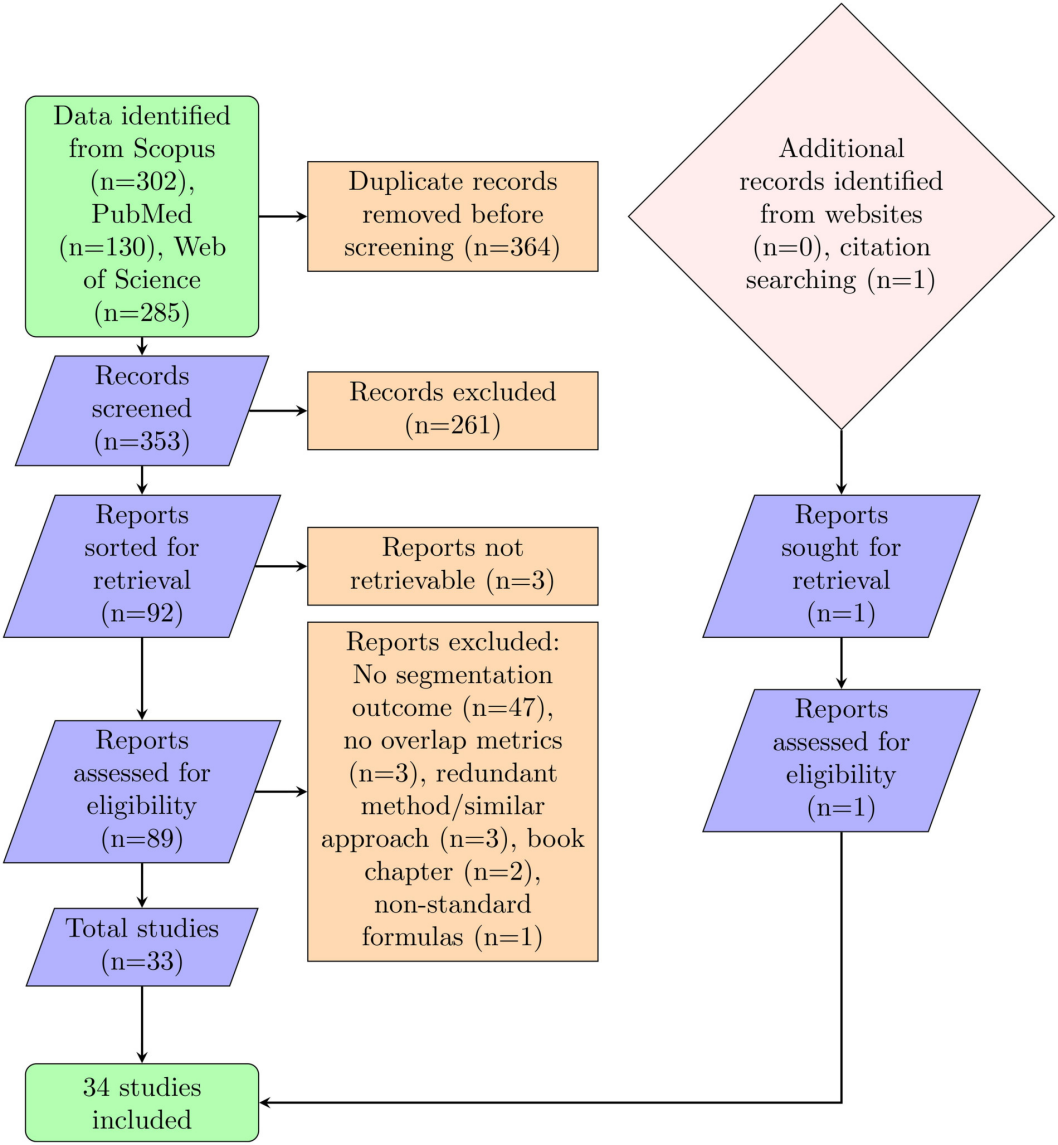
Flowchart of the search and selection process for study inclusion.

**Figure 2:**
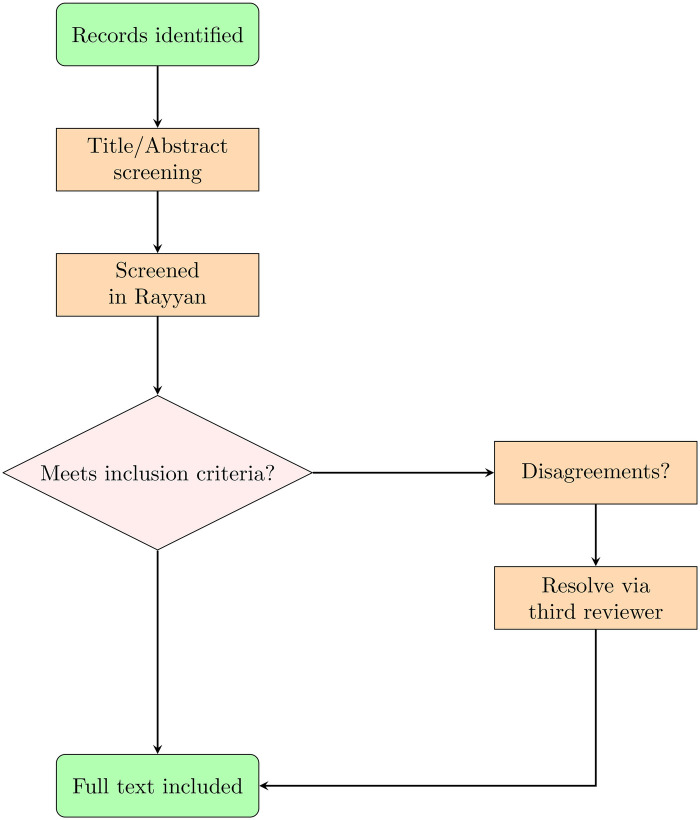
Selection process for inclusion of studies.

The PRISMA process identified 717 records across the three databases searched (Scopus = 302, PubMed = 130, Web of Science = 285) (Figure 1), and following the selection process (Figure 2), 34 were included. During title and abstract screening, seven disagreements between reviewers were identified and subsequently resolved through consultation with a third reviewer.

#### 3.1.2 Segmentation approaches for pituitary gland MRI studies

**Figure 3:**
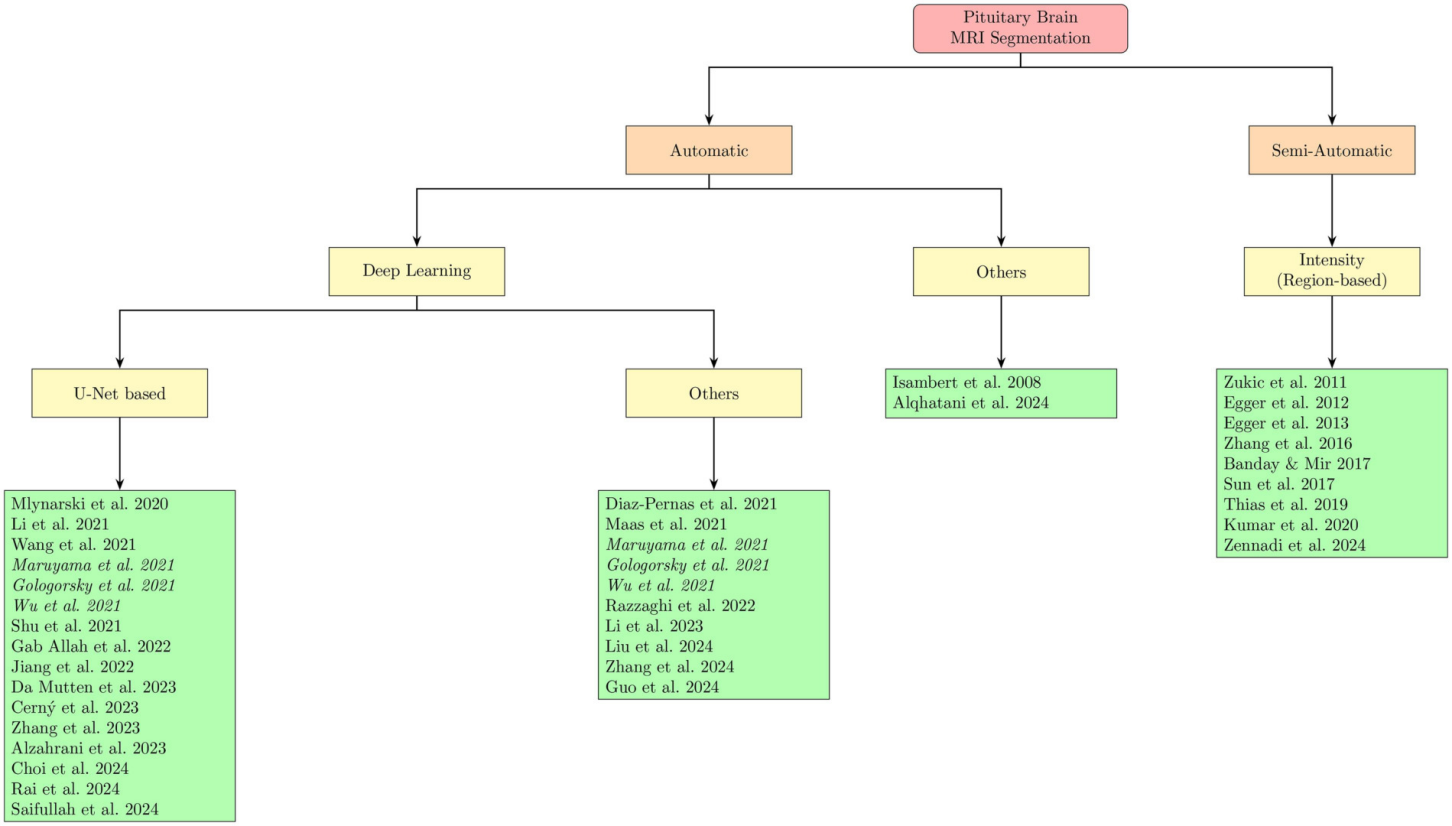
Segmentation approaches for pituitary MRI studies. A total of 16 studies employed U-Net–based architectures and 10 employed other deep learning methods. Three studies utilized both U-Net and alternative deep learning architectures and are therefore shown in *italics* and listed in both subcategories. These represent overlapping methodological approaches within the same studies and do not increase the total number of unique studies.

Segmentation techniques were classified by automation level and methods employed (Figure 3).

### 3.2 Study characteristics

A total of 34 studies out of 353 screened were included, characterized as follows:

In terms of Segmentation Approaches, 13 studies used U-Net-based models, 3 used U-Net + other DL models, **7** used other DL models, 2 applied automatic methods without DL, and **9** employed semi-automatic approaches. In terms of MRI Field Strength, 6 studies used 1.5T MRI, 2 used 3T, 5 used both 1.5T and 3T, 1 used 1T/1.5T/3T, and for 20 studies [including those using the *Cheng 2015 dataset* (16)] this information was not available. The reporting of human subject counts was inconsistent: only 13 of the 34 included studies explicitly stated the number of subjects, whereas the remainder [again, incluiding *Cheng 2015 dataset* (16)] reported dataset size in terms of images/slices or did not provide a disaggregated subject count (recorded as NR).

### 3.3 Results of individual studies

Detailed characteristics and performance metrics of the included studies are reported in Supplementary Tables S1–S4. Across all tables, information is presented on dataset source, MRI field strength, manual segmentation software (where reported), quantity of ground truth annotations (images or slices), model type, input dimensionality (2D vs. 3D), and Dice similarity coefficients.

Most of the 10 *automatic PG segmentation studies* (Supplementary Table S1) used locally acquired datasets, with some making additional use of ADNI and ABIDE datasets. MRI field strength and manual segmentation software were variably reported. Several studies evaluated multiple model architectures within the same dataset, reporting Dice scores separately for each model. Of these 10 studies, one employed a non–deep learning automatic method. Both 2D and 3D approaches were represented.

Supplementary Table S2 reports the 18 *automatic PA segmentation studies*. Datasets included local institutional data, and the Cheng 2015 dataset. Adenoma size was only reported in a small subset of studies, and MRI field strength and manual segmentation software were inconsistently documented. Some studies assessed multiple models or variants within a single cohort, with Dice scores reported per model. Among these 18 studies, one used a non–deep learning automatic approach. Likewise, both 2D and 3D segmentation strategies were used.

Supplementary Table S3 presents the three *semi-automatic PG segmentation studies*, all of which used local datasets. Reported approaches included morphological-based methods and atlas-based techniques, with both 2D and 3D implementations. Dice scores were reported for each study.

Supplementary Table S4 summarizes the six *semi-automatic PA segmentation studies* using local datasets and the Cheng 2015 dataset. Studies employed a range of contour- and graph-based techniques. Several studies reported multiple models or algorithm variants, with Dice scores provided for each. Input dimensionality was predominantly 2D, with one study reporting combined 2D and 3D use.

### 3.4 Narrative synthesis and exploratory study-level comparisons

**Figure 4:**
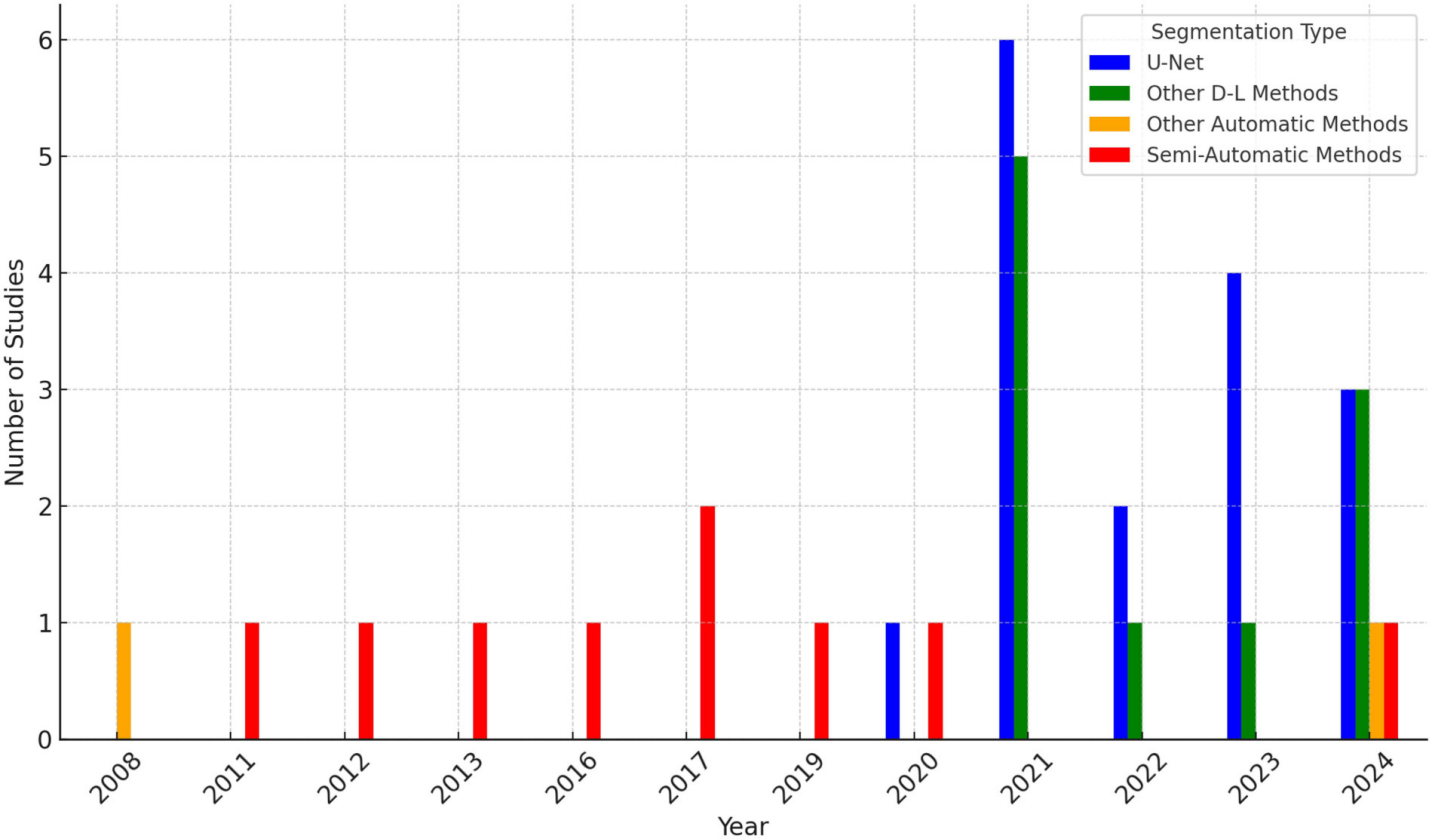
Number of segmentation studies and methodological approaches used per year. While 34 unique studies were included, this figure reflects 37 methodological entries because three studies implemented both U-Net and alternative deep learning architectures and are therefore counted in both methodological categories. These overlapping entries correspond to the same underlying studies and do not increase the total number of unique studies.

**Figure 5:**
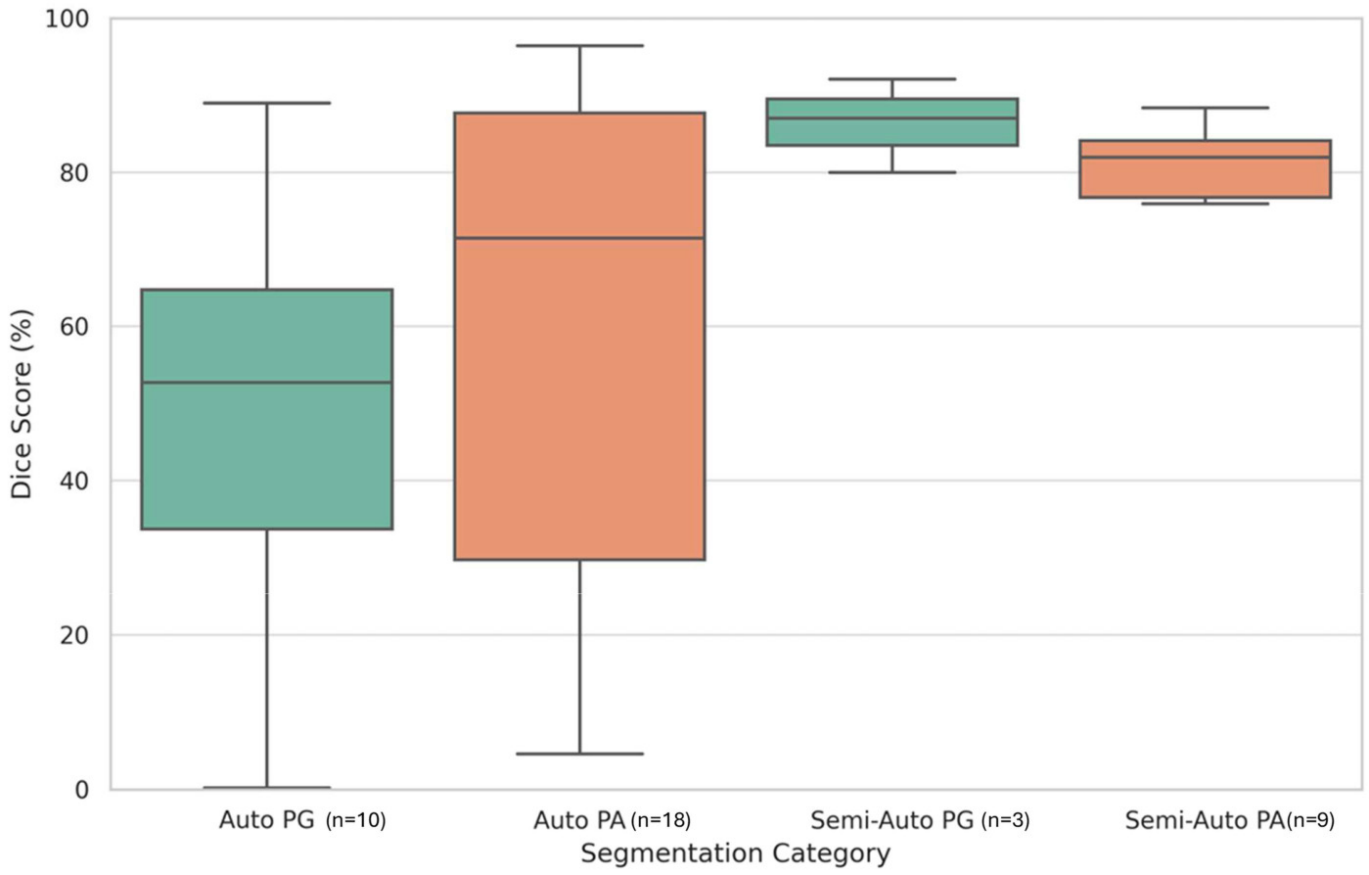
Dice score distributions for automated and semi-automated segmentation methods across PG and PA structures. Box plot horizontal lines represent the median (center), first and third quartiles (box edges), and the minimum and maximum values within 1.5× interquartile range (whiskers). Some studies reported both PG and PA while some reported multiple methods.

**Figure 6:**
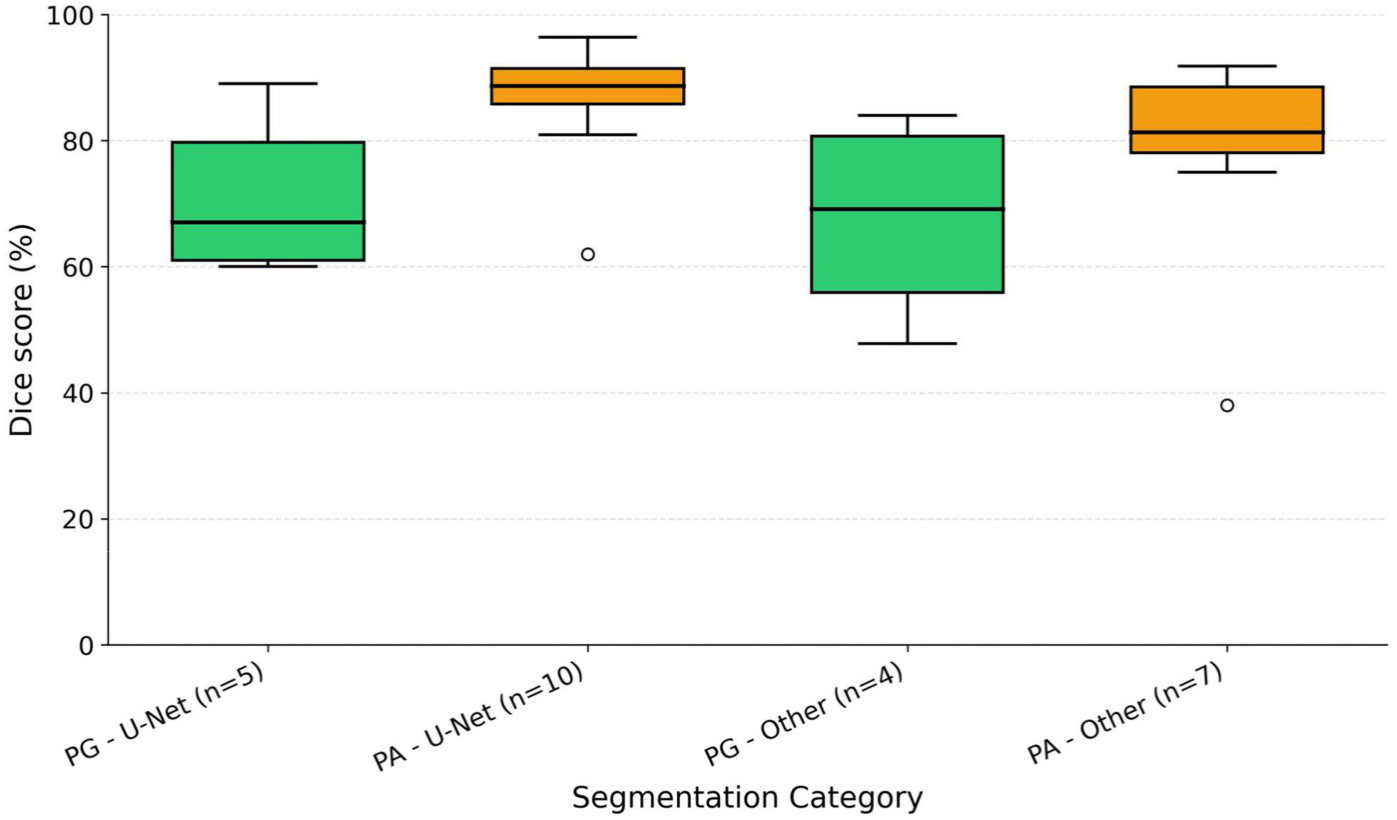
Box plot comparing Dice scores for U-Net-based and other (non-U-Net-based) deep learning models across pituitary gland (PG) and pituitary adenoma (PA) segmentation. Elements are the same as Figure 5 (dots outside the whiskers [PG-Others and PA-U-Net] represent outliers). For each study, only the highest reported Dice score was used in this comparison.

**Figure 7:**
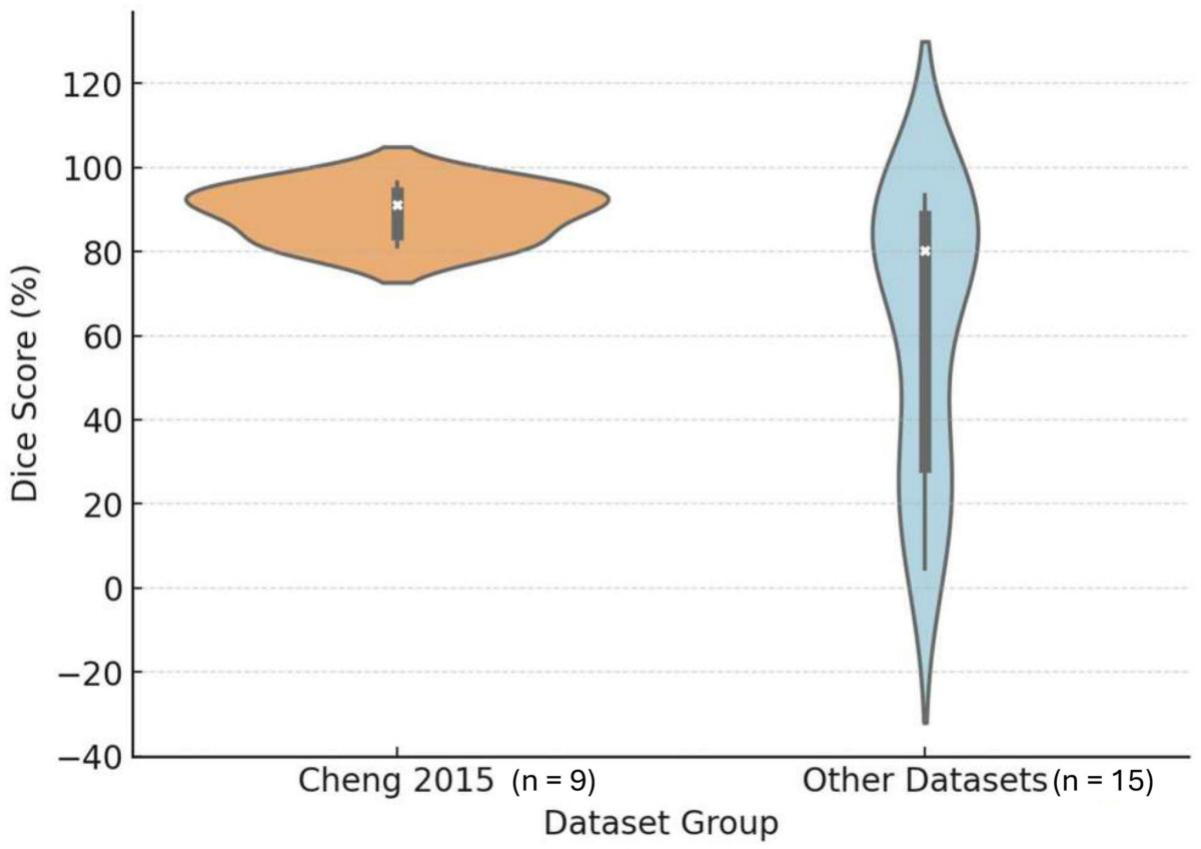
Violin plots of Dice score distribution for PA automatic segmentation: Cheng 2015 dataset vs. other datasets. The width of each violin indicates the density of data points at different Dice score values. The central white dot represents the median, the thick black bar is the interquartile range, and the thin black line shows the range within 1.5× the interquartile range.

**Figure 8:**
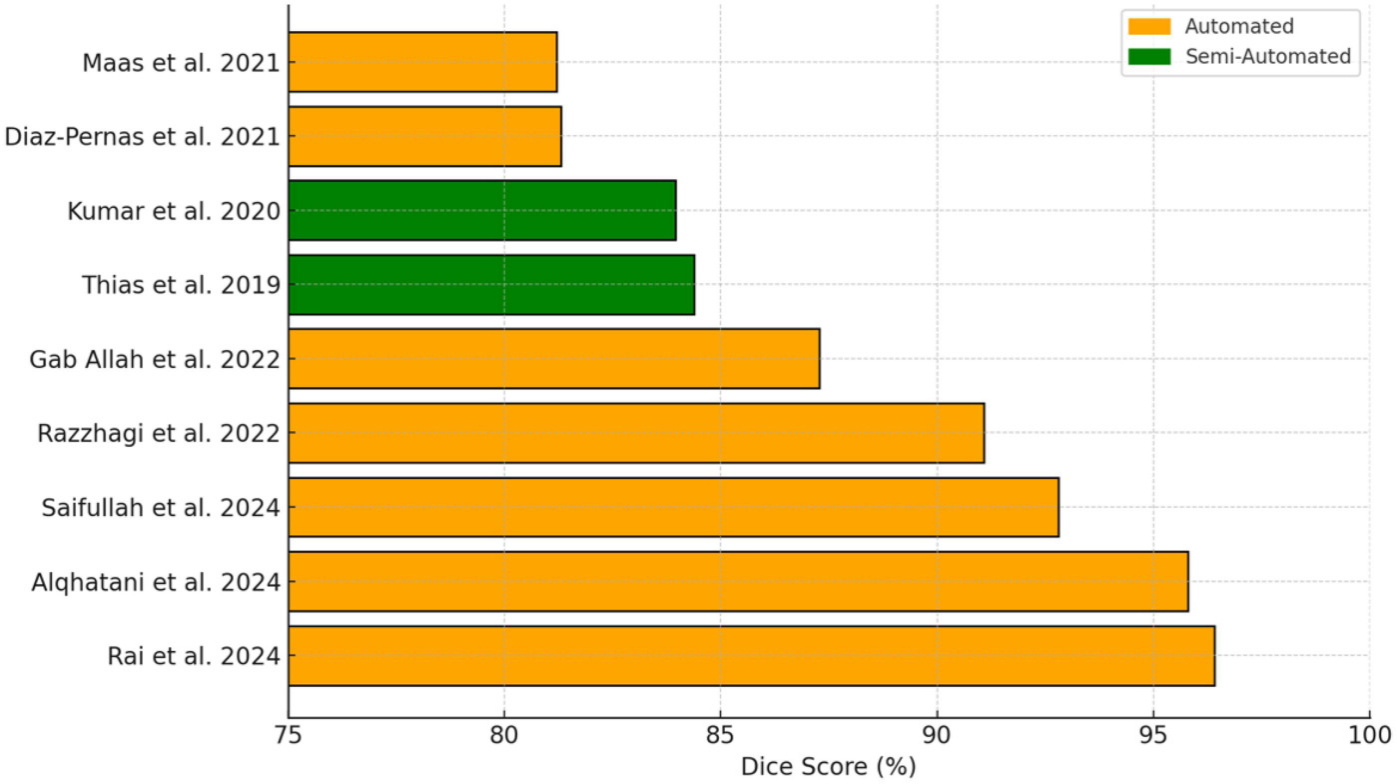
Dice scores of studies that utilized the Cheng 2015 dataset for pituitary adenoma segmentation, grouped by segmentation method. Automated models demonstrated a wider and generally higher performance range compared to semi-automated methods.

This section synthesizes quantitative trends observed across the included studies by integrating temporal patterns, segmentation methodology, model architecture, dataset usage, and reported segmentation performance. Figures 4–8 and Tables 1, 2 are interpreted collectively to provide an exploratory, study-level comparison of segmentation outcomes rather than formal meta-analytic inference.

**Table 1:** Statistical comparison of Dice scores across segmentation method groupings. Higher MW values and K-S values indicate superiority of the second comparator.

Comparison	MW	MW *p*-value	K–S statistic	K–S *p*-value
PG Auto vs. Semi-Auto	3.0	0.049*	0.800	0.070
PA Auto vs. Semi-Auto	87.0	0.158	0.524	0.085
U-Net vs. Others (PG Auto)	21.0	0.329	0.600	0.238
U-Net vs. Others (PA Auto)	41.0	1.000	0.222	0.989
Cheng vs. Other (PA Auto)	55.0	0.151	0.481	0.208
2D vs. 3D (PG Auto)	6.0	0.383	0.571	0.400
Auto vs. Semi-Auto (Cheng)	10.0	0.500	0.714	0.333

*Statistically significant (p<0.05).

MW, Mann–Whitney *U* test; K-S, Kolmogorov–Smirnov test.

**Table 2:** Reporting of demographic, tumour, and MRI acquisition characteristics across non-Cheng studies, stratified by segmentation task.

Task	Studies (*n*)	Age reported	Sex reported	Volume reported	Size/type reported	FS reported	Any MRI param	≥2 MRI params
PG	10	4	2	1	*N/A* ${}^{a}$	6	9	5
PA	12	3	4	4	4	5	8	6
PG & PA	3	2	2	0	1	3	3	3

Any MRI Param, at least one MRI acquisition parameter reported (e.g., slice thickness, voxel size, matrix, field of view, image size, slice spacing, imaging plane); FS, MRI field strength reported; ≥2 MRI Params, two or more MRI acquisition parameters reported.

${}^{a}$Adenoma size/type fields are not applicable for PG-only studies (normal pituitary gland segmentation).

#### 3.4.1 Summary of study characteristics

The number of segmentation studies per year and approaches used is shown in Figure 4. It illustrates the temporal distribution of included studies. Prior to 2020, most studies reported semi-automatic methods. From 2020 onward, studies predominantly adopted deep learning–based approaches, particularly U-Net and related architectures. Over the same period, the annual number of published segmentation studies increased, with most recent contributions focusing on fully automatic methods. The distribution of reported Dice similarity coefficients across segmentation targets and methodological categories is summarized in Figures 5, 6. Figure 5 contrasts automatic and semi-automatic methods for PG and PA segmentation, demonstrating consistently higher median Dice scores and narrower variability for semi-automatic approaches, alongside a wider performance spread among automatic methods. Figure 6 further explores performance differences within automatic segmentation by comparing U-Net–based models with other (non–U-Net-based) deep learning architectures. For studies reporting multiple architectures evaluated on the same dataset, only the highest reported Dice score per study was retained for comparative analyses. This approach ensured that each study contributed a single representative performance value, avoiding disproportionate weighting of studies that evaluated multiple models on the same cohort and enabling a fair study-level comparison across model families. Across both model categories, Dice scores were generally lower for PG segmentation than for PA segmentation. Within PG segmentation, U-Net–based models exhibited higher Dice scores and reduced dispersion relative to other deep learning approaches, whereas PA segmentation showed similar central tendencies between model families. Figure 7 compares Dice score distributions between PA studies using the Cheng dataset and those using other datasets. While Dice scores for studies using the Cheng dataset cluster more closely together than those for studies using other datasets, again the median is similar. Figure 8 summarizes reported Dice similarity coefficients from studies that utilized the *Cheng 2015* dataset for PA segmentation, grouped by segmentation method (automated vs. semi-automated). Across studies using this dataset, automatic methods span a broader range of reported Dice values, whereas semi-automatic approaches are represented by fewer studies with more clustered performance values. These findings mirror broader trends observed across all datasets, reinforcing the trade-off between automation and performance consistency.

#### 3.4.2 Non-parametric statistical comparison of segmentation performance

To complement the exploratory visual analyses, non-parametric statistical comparisons of segmentation performance are summarized in Table 1. Comparisons were conducted using the Mann–Whitney *U* (MW) test to assess differences in median Dice scores between predefined groups, alongside the Kolmogorov–Smirnov (K–S) test to compare the overall distributions of reported Dice values. The analyses included comparisons between automatic and semi-automatic segmentation methods for PG and PA, between U-Net-based and non–U-Net-based automatic models for both targets, between studies using the *Cheng 2015* dataset and those using other datasets for PA segmentation, and between two-dimensional (2D) and three-dimensional (3D) automatic PG segmentation approaches. An additional comparison assessed automatic vs. semi-automatic segmentation performance within studies using the *Cheng 2015* dataset. For each comparison, the MW test statistic and associated *p*-value are reported alongside the K–S statistic and *p*-value, providing complementary summaries of central tendency and distributional differences. A statistically significant difference was observed only for the comparison between automatic and semi-automatic PG segmentation, while all other comparisons did not reach statistical significance.

#### 3.4.3 Possible causes of heterogeneity among results

Substantial heterogeneity across studies was primarily attributable to limited reporting of MRI acquisition parameters (e.g., repetition time, echo time, flip angle). Key imaging details—including field strength, voxel size, sequence type, slice thickness, and contrast administration—were not reported in 20 of the 34 included studies. Among the remaining 14 studies, most provided only partial acquisition information, and only four studies (37–40) described MRI protocols in sufficient detail to support reproducibility.

Table 2 summarizes MRI acquisition parameter and demographics reporting across other studies that did not make use of the *Cheng 2015 dataset* (non-Cheng studies), stratified by segmentation task (PG, PA, and both PG & PA). Field strength was reported in 14 of 25 non-Cheng studies, while at least one MRI acquisition parameter was reported in 20 studies. Reporting of two or more acquisition parameters occurred in 14 studies, with variation observed across task categories. Reporting of patient demographics was also inconsistent. Nine of the 34 studies (∼26%) reported patient age or sex, and only a subset reported both. Considering the non-Cheng studies, adenoma size classification (micro, macro or giant) was reported in five studies, while adenoma type (functioning vs. non-functioning) was also reported in five studies. However, inconsistent definitions and lack of stratified performance metrics precluded comparative analysis based on these variables. Public datasets—particularly *Cheng 2015*—generally lacked demographic and clinical information altogether. In the small subset of non-Cheng studies reporting adenoma volume (n=4), Spearman correlation analysis showed no statistically significant association with Dice performance (ρ=0.32, p=0.68).

#### 3.4.4 Sensitivity analyses

Due to the heterogeneity and lack of consistent reporting (see Supplementary Tables S5–S9), sensitivity analyses were not feasible.

### 3.5 Descriptive synthesis of segmentation methodologies

This subsection provides a descriptive synthesis of segmentation methodologies and reported performance across included studies, without comparative inference or assessment of clinical superiority. By our review, the studies can be categorized into two primary segmentation approaches: automatic and semi-automatic. Within these categories, the techniques utilized varied widely with some used frequently, and some studies introduced innovative architectures and training methods to optimize performance. A single dataset with 2D slices only (*Cheng 2015 dataset*) was very commonly used.

#### 3.5.1 Automatic segmentation approaches

##### 3.5.1.1 Deep learning methods

Among the 26 studies using deep learning techniques, 16 employed U-Net-based models. The performance of these models varied, highlighting the importance of dataset quality, preprocessing, and architectural modifications. Modifications such as residual blocks (38), attention mechanisms (41), ensemble learning (42), and optimization techniques (43) helped enhance performance. While PG segmentation remained challenging due to anatomical complexity and small size, a few studies still reported high Dice scores using advanced architectures, such as DeepPGSegNet (44) (89%), though these remain exceptions rather than the norm. In contrast, PA segmentation—with larger and better-defined structural boundaries—consistently achieved higher Dice values, reaching up to 96% with EfficientNet (45).

##### 3.5.1.2 Segmentation of both PA and residual healthy PG

Several studies included both PA and residual healthy PG tissue segmentation from the same subjects in their models. These studies are significant because segmenting both structures simultaneously adds complexity due to the differences in size, shape, and tissue contrast between the PA and the normal PG (or residual healthy PG), but is also more clinically significant.

- Wang et al. employed a *Gated-shaped U-Net*, incorporating region-specific gating mechanisms to improve segmentation accuracy, achieving a Dice score of 89% for PA segmentation and 60% for the PG. The higher performance in PA segmentation was attributed to the PA’s larger size and clearer boundaries compared to the smaller and more complex anatomical structure of the residual healthy PG (40).
- Cerny et al. implemented a fully automated segmentation system using a standard *U-Net* architecture, achieving a high Dice score of 93% for PA segmentation and 61% for the residual healthy PG. Interestingly, additional pulse sequences did not enhance performance, suggesting that the core architecture was robust enough for PA segmentation without needing extra input modalities (37).

##### 3.5.1.3 Reported deep learning architectures and performance ranges

For *PG segmentation*, the consistent challenge across studies has been the small size and indistinct anatomical boundaries of the pituitary gland. Nonetheless, several approaches have yielded promising results. Alzahrani et al. adopted a multi-modal approach, integrating CT and MRI contours into U-Net frameworks, which led to a Dice score of 67% (46). Mlynarski et al. demonstrated that minor architectural modifications to the conventional U-Net, such as having one segmentation layer per class trained on three planes with majority voting, and allowing for one-voxel mismatch to assess the performance, can improve segmentation performance metrics on small structures, reporting a Dice score of 79% (47). Building upon architectural enhancements, Gologorsky et al. implemented ensemble learning with a suite of 3D U-Net variants, achieving up to 79% Dice score by leveraging diverse model outputs (42). Meanwhile, Liu et al. introduced attention mechanisms into their architecture via the Channel Attention Long-Short-Term-Memory (CALN) model, combining Long-Short-Term-Memory (LSTM) and channel attention to enhance fine-grained spatial detail, achieving 84% (41). The highest performance was observed in the study by Choi et al., whose *DeepPGSegNet* achieved a Dice score of 89% despite being trained on a relatively narrow age cohort, underscoring the potential of deeper, well-optimized U-Net architectures for small structure segmentation (44).

In contrast, *PA segmentation* presents a larger structural target, especially for macroadenomas and giant PAs but still benefits from the adaptability of U-Net-based models. Several studies emphasized architectural optimization to handle variations in adenoma size and morphology. Shu et al. used the dynamic, self-configuring nnU-Net framework to accommodate diverse tumor types, achieving up to 85% Dice, with improved performance on larger lesions (39). Similarly, Wang et al. employed a gated-shaped U-Net incorporating spatial attention to segment both PA and residual healthy PG tissue, achieving 89% Dice for PA (40). Jiang et al. addressed feature scaling challenges through a modified U-Net with cross-layer connections, resulting in an 88% Dice score (48). Cerný et al. demonstrated that even a conventional 3D U-Net, when trained on a relatively large and well-curated dataset (521 MRI scans from 493 patients), can achieve high segmentation performance—reporting a Dice score of 93% (37), the highest among reviewed studies that did not utilize the *Cheng 2015 dataset* (16) 2D slices.

From a multi-scale learning perspective, Zhang et al. introduced parallel dilated convolutions and attention modules within their Parallel Dilated Convolutional (PDC) U-Net to enhance boundary representation, while Zhang et al. developed Multiscale Residual Network (MSR-Net) with dual decoding paths, attaining Dice scores of 88% and 89%, respectively (49, 50).

Other studies tackled PA segmentation through model regularization and optimization strategies. Saifullah et al. incorporated Particle Swarm Optimization (PSO) into a CNN-U-Net hybrid to fine-tune hyperparameters such as learning rate and dropout, yielding 92% (43). Li et al. used a residual U-Net to enhance feature extraction across scales, achieving 80% (38). Wu et al, in a comparative benchmarking study, observed that U-Net underperformed (7%) relative to deeper architectures such as DeepMedic (29%), illustrating the limitations of basic U-Net models when data is sparse or heterogeneous (51). The highest Dice score was reported by Rai et al., who designed a dual-headed UNet-EfficientNet model capable of simultaneous classification and segmentation, and further improved performance through post-processing using connected component labeling, reaching 96% (45).

##### 3.5.1.4 Other deep learning approaches

Several studies explored deep learning methods beyond the commonly used U-Net architecture, yielding diverse results in PA and PG segmentation. Guo et al. also addressed simultaneous segmentation of PA and the residual healthy PG using a Mask R-CNN–based framework, reporting Dice scores of 75% for PA and 48% for PG, showcasing performance of non–U-Net deep learning approaches to dual-structure pituitary segmentation (52). As for single segmentation task, the multiscale feature integration demonstrated by Diaz-Pernas et al. and the progressive refinement strategy of cfVB-Net in Li et al. in PA segmentation, illustrate how architectural choices can directly address the challenges of segmenting small and variable anatomical structures obtaining Dice scores of 81% and 87% respectively (53, 54). Gologorsky et al. further highlighted the benefits of ensemble learning, showing how the aggregation of complementary model outputs can yield more robust segmentation outcomes in complex regions like the sellar and para-sellar space achieving a Dice score of 79% (42).

##### 3.5.1.5 Reported performance of U-Net and non–U-Net architectures

*Performance metrics:* While U-Net-based architectures delivered strong results—reaching Dice scores as high as 96% in PA segmentation (45) and 89% in PG segmentation (44)—several non-U-Net approaches produced competitive outcomes. For example, the multiscale method by Diaz-Pernas et al. (54) achieved 81%, and the modified QuickNAT model by Maas et al. (55) reached 81%. However, not all alternative architectures matched this performance; Wu et al. (51), using only 155 training cases, reported Dice scores below 40% across several tested models, underscoring the importance of data volume and quality.

*Computational requirements:* Architectures such as QuickNAT (56) and the multiscale CNN (54) introduced greater computational demands compared to baseline U-Net models due to their pretraining and structural complexity. While U-Net’s design is known for its efficiency and ease of implementation (57), these advanced methods often rely on more complex layer hierarchies or ensemble strategies. Nevertheless, the added architectural complexity did not consistently translate into superior segmentation performance; modified QuickNAT (55) and the multiscale CNN (54) achieved Dice scores around 81%, comparable to conventional U-Net models. However, such designs may still offer advantages in tasks requiring finer boundary delineation or handling of variable tumor morphologies.

*Application scenarios:* A recurring theme among these alternative approaches is their alignment with specialized use cases. Li et al. (53) integrated segmentation with radiomics to predict the Ki67 proliferation index—a clinically relevant biomarker—highlighting the potential of tailored architectures like cfVB-Net in personalized diagnostics. Similarly, Gologorsky et al. (42) addressed broader brain region segmentation using diverse volumetric models, reflecting a different operational focus from typical lesion-targeted pipelines.

In summary, while U-Net and its derivatives remain dominant in pituitary segmentation due to their reliability and flexibility, other deep learning strategies offer valuable alternatives in specific scenarios. These models expand the methodological toolkit for handling diverse data characteristics, enhancing performance where traditional architectures may encounter limitations.

##### 3.5.1.6 Other (non–deep learning) automatic methods

A few studies explored non-deep learning automatic methods. Isambert et al. used an atlas-based approach (ABAS), developed from synthetic data (58), for PG segmentation and achieved a low Dice score (30%) due to anatomical variability (59). In contrast, Alqhatani et al. applied Fuzzy C-Means (FCM) clustering with preprocessing enhancements for PA, achieving 95%. These approaches show potential but remain highly dependent on structure type and dataset conditions (60).

##### 3.5.1.7 Comparison with deep learning approaches

The Dice score of 30% reported by Isambert et al. (59) using the ABAS atlas-based method highlights the limitations of non-deep learning approaches in segmenting small structures like the PG, where deep learning models such as U-Net have achieved up to 79% (47) and 89% (44). While multi-atlas methods may perform comparably to deep learning in other contexts (61), ABAS’s synthetic-anatomy approach performance remains low. Alqhatani et al. (60) achieved a high Dice score of 95% for PA segmentation using FCM clustering and enhancement techniques, nearly matching the 96% by Rai et al. (45) using a UNet-EfficientNet model. However, both relied on the same dataset (16), potentially inflating performance.

#### 3.5.2 Semi-automatic segmentation

Intensity (region-based) segmentation approaches have proven effective in PG and PA delineation, offering an alternative to deep learning when computational resources or annotated datasets are limited. Zhang et al. and Banday & Mir combined wavelet transforms with mathematical morphology for PA segmentation, achieving Dice scores of 87% and 92%, respectively (62, 63). For PG, Zennadi et al. (64) used SPM12 to create probabilistic atlases from young adult female MRIs, reaching 80% accuracy, though demographic bias (65–67) was noted.

Early innovations by Zukic et al. and Egger et al. applied balloon inflation, Grow-cut, and graph-based methods for PA segmentation, achieving scores between 75% and 81% (68–70). Sun et al. combined random walk initialization with active contours to enhance segmentation accuracy (88%) (71). Meanwhile, Thias et al. and Kumar et al. reported strong results (up to 84%) using active contour and edge-based approaches on the *Cheng 2015 dataset* (72, 73). These methods demonstrate that minimal-intervention tools can still yield reliable outcomes for PA segmentation, especially for structurally distinct or homogeneous lesions.

## 4 Discussion

This systematic review was designed to characterize trends in pituitary segmentation methodologies and reported performance rather than to derive a pooled estimate of segmentation accuracy. A formal meta-analysis was not undertaken due to heterogeneity in study populations, repeated evaluation of multiple models on overlapping datasets, and inconsistent reporting of sample size and variance.

### 4.1 Comparative analysis

Automatic methods, particularly deep learning-based approaches, achieved the highest reported Dice scores for PA segmentation but showed greater variability across studies (Figure 5). Semi-automatic techniques, by contrast, yielded more consistent results with higher median Dice values, particularly for PG segmentation, where user-guided refinement appears advantageous. Statistical analysis confirmed this trend, with semi-automatic methods achieving significantly higher Dice scores for PG segmentation (*p* = 0.049, Mann–Whitney *U* test) (see Table 1). These differences likely reflect structural and imaging challenges—automatic models tend to perform well for larger, well-defined adenomas, whereas semi-automatic methods provide greater precision for small and anatomically variable structures like the PG. Despite this, U-Net variants demonstrated strong and adaptable performance across both PG and PA (Figure 6), supporting their continued development for integrated clinical workflows.

A key distinction between these approaches lies in user interaction. Automatic models operate independently once trained, offering fast and reproducible inference across large datasets—an advantage for high-throughput applications. In contrast, semi-automatic methods such as *GrowCut* (68) and region-based active contour techniques rely on user input for initialization or boundary correction. While this dependence can introduce inter-operator variability, it can also enhance segmentation accuracy in cases with indistinct anatomical borders or poor image contrast.

In terms of computational demand, deep learning requires extensive training resources, as highlighted by Wu et al. and Li et al. (51, 53). Semi-automatic techniques, such as balloon inflation (70) and graph-based active contouring (71), are computationally lighter and suitable for low-resource settings.

Clinically, automated methods suit high-throughput needs—provided failure rates are low, while semi-automatic tools remain relevant where expert guidance is feasible or data are limited. Region-based methods like those by Thias et al. and Kumar et al. still deliver strong performance for focused tasks (72, 73). Taken together, these considerations illustrate a practical workflow trade-off: fully automatic approaches prioritize speed and scalability with minimal user interaction, whereas semi-automatic methods require additional user input but may offer greater control and reliability in anatomically challenging cases. Nevertheless, a key factor determining the clinical adoption of automated segmentation workflows—highlighted across multiple studies (37, 39, 45, 53, 74–77)—is the extent to which models are trained and validated on external, diverse datasets. In this review, none of the included studies reported the results of external validation on independent cohorts, meaning that the generalizability of current pituitary segmentation models beyond their development data remains untested. Without such validation, even high-performing models may lack robustness across scanners, institutions, and patient populations.

#### 4.1.1 Summary of key methodological trends

Across the reviewed literature, several consistent methodological patterns emerged. First, deep learning approaches—particularly U-Net–based architectures—dominate recent studies and generally achieve the highest reported Dice scores, especially for pituitary adenoma segmentation. Second, semi-automatic approaches remain competitive in scenarios involving small or anatomically complex structures such as the normal pituitary gland, where user-guided refinement can improve boundary accuracy. Third, many studies demonstrate strong performance under controlled experimental conditions but rely on single-centre datasets or benchmark datasets such as the *Cheng 2015 dataset*. Finally, several recurring limitations were identified across studies, including limited reporting of MRI acquisition parameters, inconsistent documentation of patient demographics and tumour characteristics, and the absence of external validation on independent cohorts. Collectively, these patterns highlight both the methodological progress in automated pituitary segmentation and the key challenges that must be addressed before routine clinical deployment.

### 4.2 Challenges and limitations

Despite promising advances, the studies reviewed exhibit several recurring limitations that impact the interpretability, comparability, and clinical applicability of segmentation models for PG and PA.

One notable challenge in assessing performance is the inconsistent reporting of tumour characteristics—particularly the size of PAs. Many studies, such as (48, 78), reported high Dice scores for PA segmentation but did not specify whether the tumors were micro, macro, or giant adenomas (Table 2). Since larger structures tend to yield higher Dice scores due to clearer anatomical boundaries (61) and lower surface-to-volume ratios, this lack of stratification limits the ability to fairly assess model performance across different tumour complexities. By contrast, studies such as (38, 39) provided more granular tumor size classifications, offering a more nuanced understanding of model capabilities across lesion types. Correspondingly, an exploratory Spearman correlation analysis performed on the small subset of non-Cheng studies reporting PA volume did not identify a statistically significant association between tumour volume and Dice performance (Table 2), highlighting the limited statistical power and sparse reporting of volumetric data.

Furthermore, the overwhelming reliance on Dice or Jaccard scores—without complementary metrics like Average Symmetric Surface Distance (ASSD)—restricts insight into boundary accuracy, particularly in smaller or irregularly shaped lesions. Although Dice is widely used, the Jaccard index provides a stricter overlap evaluation: $J=\frac{\left|A\cap B\right|}{\left|A\cup B\right|}$; $D=\frac{2|A\cap B|}{\left|A|+|B\right|}$. These metrics are mathematically related as $D=\frac{2J}{1+J}$ and $J=\frac{D}{2-D}$ (79, 80). Jaccard theoretically controls Dice (81) and offers better numerical discrimination for high overlaps. In simple terms, Dice and Jaccard scores measure how closely an automated segmentation (predictions) matches the region outlined by a human expert (manual ground truth), with values ranging from 0 to 1 and higher values indicating greater spatial overlap between the two. From a clinical perspective, better overlap may support more reliable volumetric assessment, treatment planning, and follow-up evaluation.

Data limitations also remain a common concern. The *Cheng 2015 dataset* (16)—used by ∼26% of included studies - comprises 2D axial slices rather than full 3D volumes, which limits spatial continuity and restricts development of volumetric segmentation models. In addition, the images represent tumor-containing slices only, meaning that the model is consistently exposed to positive cases and does not encounter normal or tumor-free regions. This design simplifies segmentation tasks but may overestimate real-world performance, where non-lesional slices are common (Figure 7). Furthermore, the dataset lacks accompanying demographic and clinical information (e.g., age, sex, tumor subtype, or hormonal status), and all data originate from a single imaging center using equipment available at the time of acquisition. Although the dataset includes 233 patients overall, patient counts are not stratified by tumor class, making it impossible to determine how many patients contributed pituitary adenoma data. These factors together underscore that while the dataset is instrumental for method development, performance on it may not fully generalize to multi-institutional clinical settings. While some studies, such as Cerný et al. (37), leveraged relatively large (*n* = 521) internal datasets, most lacked external validation. High-performing models like those in Rai et al. (45) and Alqhatani et al. (60) achieved Dice scores above 95% using the Cheng dataset, yet their results were not validated on independent clinical data. On the other end of the spectrum, Wu et al. (51) demonstrated how segmentation models trained on relatively small datasets (*n* = 155) struggled to perform consistently across architectures, with Dice scores dropping below 40%. These examples underscore the importance of both data diversity and cross-institutional validation when evaluating generalizability. While the *Cheng 2015 dataset* remains a valuable open-access benchmark that has facilitated comparability across studies, several contextual considerations are important.

While semi-automatic methods present an alternative that often requires less data and computational power, they are not without limitations. Algorithms such as Grow-cut (68), random walk-based models (71), and balloon inflation techniques (70) all require varying degrees of user input. Manual initialization introduces inter-operator variability, reducing reproducibility—especially for less experienced users. Additionally, while methods like those proposed by Banday & Mir (63) and Kumar et al. (72) showed high Dice scores, their application remains constrained to relatively homogeneous tumor presentations, with less evidence supporting their effectiveness in complex or ambiguous anatomical cases.

Finally, atlas-based segmentation approaches, while historically useful even in small brain structures such as the piriform cortex (82), also face limitations when applied to PG. Isambert et al. (59) employed a fully automatic pipeline using atlas-based automatic segmentation software (ABAS)—developed from a synthetic MRI (58). While this approach enabled high anatomical consistency during development, the use of artificially generated data may have introduced domain discrepancies when applied to real clinical images—potentially contributing to the relatively low Dice score of 30% reported for PG segmentation. In contrast, Zennadi et al. (64) (Dice score of 80%) used a maximum probability atlas (MPA) within the SPM environment, requiring multi-step normalization and manual parameter tuning—characteristics that justify its classification under semi-automatic methods.

Across the reviewed literature, several methodological strengths are evident. Many studies demonstrate that modern deep learning architectures—particularly U-Net derivatives—can achieve high segmentation performance under controlled conditions, especially for PA with clear anatomical boundaries. Semi-automatic approaches consistently report stable performance for both PG and PA segmentation (Figure 5 and Supplementary Tables S3, S4), highlighting their robustness in scenarios with limited data or complex anatomy. In addition, the increasing use of standardized performance metrics, most commonly the Dice similarity coefficient, enables broad comparison across studies.

However, these strengths are counterbalanced by recurring limitations. Performance gains are frequently demonstrated on single-center or benchmark datasets with limited clinical variability, most notably the Cheng dataset. Reporting of key acquisition parameters, patient demographics, and tumour characteristics remains sparse, restricting reproducibility and limiting the ability to contextualize reported Dice scores. Moreover, the absence of external validation across all included studies constrains assessment of generalisability, particularly across scanners, institutions, and patient populations.

Taken together, the reviewed studies demonstrate that while segmentation accuracy under experimental conditions is increasingly high, methodological and reporting limitations currently hinder translation into routine clinical practice. Addressing these gaps—particularly in dataset diversity, protocol transparency, and validation strategy—represents a necessary step toward reliable clinical deployment. Collectively, these challenges emphasize the need for larger, diverse, and well-annotated datasets; standardized reporting of PG & PA features; inclusion of multimodal clinical data; and external validation. As segmentation models advance, these methodological improvements will be critical to ensuring their reliability, generalizability, and eventual clinical adoption.

#### 4.2.1 Study limitations

In this systematic review, a noteworthy limitation is the time interval between the last database search (October 2024) and the completion of this manuscript (July 2025). Given the rapid pace of developments in medical image segmentation and deep learning, it is possible that additional studies have since been published that further advance the field. In addition, a quantitative meta-analysis was not feasible due to the heterogeneity and limitations in the reporting of MRI acquisition parameters, dataset characteristics, and PG & PA features.

### 4.3 Future directions

Future work should focus on developing generalisable models for PG and PA segmentation across diverse imaging protocols and populations, supported by large, multi-institutional annotated datasets. Integrating multimodal information—such as clinical or endocrine markers—may enhance diagnostic relevance and bridge the gap between imaging and functional assessment. Standardised benchmarking frameworks and rigorous external validation are essential to enable fair comparison across methods and to support clinical translation.

From a clinical implementation perspective, future studies should be designed as prospective or retrospective clinical validation studies that explicitly account for the methodological limitations identified in this review, including variability in imaging protocols, limited reporting of acquisition parameters, and inconsistent documentation of patient and tumour characteristics. Addressing these factors within study design would facilitate more robust evaluation of model reliability and generalisability.

In neuroradiological workflows, reliable automated segmentation could support time-efficient volumetric assessment, improved longitudinal monitoring of tumour growth or treatment response, and more consistent preoperative evaluation of tumour extent and residual healthy pituitary tissue. Integration of segmentation tools into routine reporting or surgical planning systems may also reduce inter-observer variability and support decision-making in complex cases. Demonstrating these tangible workflow benefits through clinically focused evaluation will be critical for broader adoption.

## 5 Conclusion

This review set out to evaluate the reliability and current readiness of segmentation methods for PG and PA on MRI. Deep learning models—particularly U-Net variants—have shown strong performance, with reported Dice scores reaching 89% for PG and 96% for PA, indicating high performance and clinical promise.

Many studies lacked tumor size stratification or external validation, limiting the generalizability of reported performance. Our findings emphasize that while automated segmentation methods for the PG and PA show promising performance, variability in datasets and validation practices currently constrains their clinical translation. Semi-automatic methods remain relevant in contexts where data or computational resources are limited, achieving Dice scores above 80% in focused clinical applications.

Overall, automated segmentation tools for PG and PA are increasingly high-performing and scalable, but their adoption will depend on improved dataset diversity, external validation, and reported PG & PA features, demonstrating feasibility and reliability. Addressing these challenges is essential for real-world deployment in diagnosis, surgical planning, and longitudinal monitoring of pituitary disorders. Future work may enable formal meta-analytic pooling once standardized reporting of subject counts, variance measures, and independent test cohorts becomes more widespread in pituitary imaging studies.

## Data Availability

The original contributions presented in the study are included in the article/Supplementary Material, further inquiries can be directed to the corresponding author.
