# Supplementary material for: Systematic review of pituitary gland and pituitary adenoma automatic segmentation techniques in magnetic resonance imaging
Source: Front Radiol. 2026 Apr 10;6:1737075. doi: 10.3389/fradi.2026.1737075 (PMC13106132; doi:10.3389/fradi.2026.1737075)
Supplement: Supplementary file 1 [file Datasheet1.pdf]

Supplementary Material

This file contains the supplementary tables referenced in the manuscript.

—

Table S1. Outcome of Automatic PG Segmentation Studies.

Study	Dataset Used	Subjects (n)	MRI Strength (Tesla)	Manual Segmentation Software	Ground Truth (Images/slices)	Number of Models Used	Model Number	Model Description	2D vs. 3D	Dice
[1]	Local	11	1.5	N/A	11	1	1	ABAS	2D	30.00
[2]	Local	NR	N/A	N/A	29	1	1	Modified 2D U-Net (OVMA)	2D	79.70
[3]	Local	163	3.0	ITK-Snap	213	1	1	Gated-shaped U-Net	2D	60.00
[4]	ADNI	NR	1.5 & 3.0	MATLAB Image Labeler	450	6	1	AlexNet	2D	46.86
							2	GoogleNet		42.39
							3	ResNet50		47.32
							4	SegNet		49.96
							5	SegNet with VGG16 weighting factor		58.65
							6	U-Net		0.19
[5]	ABIDE	NR	N/A	3D Slicer	333	7	1	UNET3D	2D	79.10
							2	VNET		77.10
							3	CONDSEG		79.30
							4	OBELISK 96		75.20
							5	OBELISK 144		74.50
							6	UNETR		72.00
							7	Ensemble		79.60
[6]	Local	521	1.5 & 3.0	ITK-Snap	521	1	1	U-Net	3D	61.10
[7]	Local	NR	N/A	N/A	32	3	1	U-Net-MRIu	3D	35.00
							2	U-Net-MRIeCT		32.00
							3	U-Net-MRIeMRI		67.00
[8]	Local	153	3.0	ITK-Snap	153	1	1	3D U-Net	3D	89.00
[9]	Local	NR	3.0 & 1.5	N/A	195	1	1	CALN	2D	84.02
[10]	Local	275	3.0 & 1.5	ITK-Snap	2586	1	1	Mask R-CNN	2D	47.77

2D: Two-Dimensional; 3D: Three-Dimensional; ABAS: Atlas-based automatic segmentation software; ABIDE: Autism Brain Imaging Data Exchange; ADNI: Alzheimer's Disease Neuroimaging Initiative; CALN: Channel Attention (Long-Short-Term-Memory) Network; CONDSEG: Conditional Segmentation; MRI: Magnetic Resonance Imaging; N/A: Not available; NR: not reported/unclear; OBELISK: Object Boundary Extraction using Learned Image Structures; OVMA: One Voxel Mismatch Allowed; PG: Pituitary Gland; Subjects (n): number of human participants when explicitly reported; UNET3D: 3D version of U-Net; UNETR: U-Net Transformer; VNET: V-Net.

Table S2. Outcome of Automatic PA Segmentation Studies

Study	Dataset Used	Subjects (n)	Adenoma Size	MRI Strength (Tesla)	Manual Segmentation Software	Ground Truth (Images/slices)	Number of Models Used	Model Number	Model Description	2D vs. 3D	Dice
[11]	Cheng 2015	NR	N/A	N/A	N/A	930	1	1	Multiscale CNN	2D	81.30
[12]	Cheng 2015	NR	N/A	N/A	N/A	930	1	1	Modified QuickNAT	2D	81.20
[13]	Local	185	Micro-PA & Macro-PA	1.5 & 3.0	MITK	185	1	1	Res U-Net	3D	80.93
[3]	Local	163	N/A	3.0	ITK-Snap	213	1	1	Gated-shaped U-Net	2D	89.80
[14]	NTUH	155	N/A	N/A	N/A	155	5	1	V-Net_dropout	2D	27.00
								2	Deconvnet		38.00
								3	U-Net		7.00
								4	PSPNet4		24.00
								5	DeepMedic		29.00
[15]	Local	243	Micro, Macro & Giant PA	1.5	ITK-Snap	243	2	1	U-Net-All type PA	3D	80.30
								2	U-Net-Primary NFPA		85.30
[16]	Cheng 2015	NR	N/A	N/A	N/A	930	1	1	Edge U-Net	2D	87.28
[17]	Local	NR	N/A	N/A	LabelMe	500	1	1	Modified U-Net	2D	88.87
[18]	Cheng 2015	NR	N/A	N/A	N/A	930	1	1	Multimodal CNN	2D	91.08
[19]	Local	1214	Micro, Macro & Giant PA	N/A	ITK-Snap	155	1	1	cfVB-Net	3D	87.70
[20]	Local	213	Micro, Macro & Giant PA	1, 1.5 & 3.0	N/A	213	2	1	U-Net-Preoperative	2D	62.00
								2	U-Net-Postoperative		4.60

[6]	Local	521	N/A	1.5 & 3.0	ITK-Snap	521	1	1	U-Net	3D	93.40
[21]	Local	38	N/A	N/A	LabelMe	2000	1	1	PDC U-Net	2D	88.45
[22]	Cheng 2015	NR	N/A	N/A	N/A	930	1	1	EfficientNet	2D	96.41
[23]	Cheng 2015	NR	N/A	N/A	N/A	930	1	1	FCM	2D	95.80
[24]	Local	NR	N/A	N/A	LabelMe	2105	1	1	MSR-Net	2D	89.34
[25]	Cheng 2015	NR	N/A	N/A	N/A	930	1	1	Modified CNN-U-Net framework	2D	92.05
[10]	Local	275	N/A	3.0 and 1.5	ITK-Snap	1617	1	1	Mask R-CNN	2D	74.97

2D: Two-Dimensional, 3D: Three-Dimensional, cfVB-Net: coarse-to-fine VB-Net, CNN: Convolutional Neural Network, FCM: Fuzzy C-Means, Giant PA: Giant Adenoma, Macro-PA: Macroadenoma, Micro-PA: Microadenoma, MRI: Magnetic Resonance Imaging, MSR-Net: Multi-Scale Residual Network, N/A: Not available, NR: not reported/unclear, NFPA: Non-Functioning Pituitary Adenoma, NTUH: National Taiwan University Hospital, PA: Pituitary Adenoma, PDC U-Net: Parallel Dilated Convolution U-Net, PSPNet4: Pyramid Scene Parsing Network version 4, Res U-Net: Residual U-Net Subjects (n): number of human participants when explicitly reported

Table S3. Outcome of Semi-Automatic PG Segmentation Studies

Study	Dataset Used	Subjects (n)	MRI Strength (Tesla)	Manual Segmentation Software	Ground Truth (Images/slices)	Number of Models Used	Model Number	Model Description	2D vs. 3D	Dice
[26]	Local	NR	N/A	N/A	10	1	1	MB	2D	87.00
[27]	Local	NR	1.5	N/A	10	1	1	MB	2D	92.10
[28]	Local	26	1.5	SPM	100	1	1	MPA	3D	80.00

2D: Two-Dimensional, 3D: Three-Dimensional, MB: Morphological Based, MPA: Maximum Probability Atlas, MRI: Magnetic Resonance Imaging, N/A: Not available, NR: not reported/unclear, PG: Pituitary Gland, SPM: Statistical Parametric Mapping Subjects (n): number of human participants when explicitly reported

Table S4. Outcome of Semi-Automatic PA Segmentation Studies

**Study**	**Dataset Used**	**Subjects (n)**	**Adenoma Size**	**MRI Strength (Tesla)**	**Manual Segmentation Software**	**Ground Truth (Images/slices)**	**Models Used**	**Model Number**	**Model Description**	**2D vs. 3D**	**Dice**
[29]	Local	NR	N/A	N/A	N/A	10	1	1	Balloon inflation-based	2D	75.92
[30]	Local	NR	N/A	1.5	MeVisLab	10	1	1	Grow-Cut in Slicer	2D & 3D	81.97
[31]	Local	NR	N/A	1.5	N/A	10	2	1	Graph-based	2D	77.50
								2	Balloon inflation-based		75.90
[32]	N/A	23	N/A	N/A	ITK-Snap	23	1	1	Random Walk & Graph-Cut-Based Active Contour	2D	88.36
[33]	Cheng 2015	NR	N/A	N/A	N/A	915	3	1	SAC	2D	83.76
								2	MACWE		83.76
								3	MGAC		84.39
[34]	Cheng 2015	NR	N/A	N/A	N/A	930	1	1	Edge-based contouring	2D	83.95

2D: Two-Dimensional, 3D: Three-Dimensional, MACWE: Morphological Active Contour Without Edge, MGAC: Morphological Geodesic Active Contour, MRI: Magnetic Resonance Imaging, N/A: Not available, NR: not reported/unclear, PA: Pituitary Adenoma, SAC: Snake Active Contour, Subjects (n): number of human participants when explicitly reported

Table S5. Automatic PG Segmentation Study Characteristics : Demographics, Imaging, and Preprocessing Details

**Study**	**Male (%)**	**Female (%)**	**Age**	**Mean PG Volume**	**Imaging Sequence**	**Preprocess**	**Augmentation**	**Post-process**
Isambert et al. 2008	N/A	N/A	47***	N/A	T1 Axial 3mm	No	No	No
Mlynarski et al. 2020	N/A	N/A	N/A	N/A	N/A	Yes	No	No
Wang et al. 2021	35	65	38.64 +/- 12.14**	N/A	N/A	No	No	No
Maruyama et al. 2021	N/A	N/A	N/A	N/A	N/A	No	Yes	No
Gologorsky et al. 2022	N/A	N/A	7-64*	N/A	N/A	Yes	Yes	Yes
Cerny et al. 2023	50	50	N/A	N/A	CE-T1 Coronal	Yes	Yes	No
Alzahrani et al 2023	N/A	N/A	N/A	N/A	T1 Axial 2mm slice thickness 1mm spacing	No	No	No
Choi et al. 2024	35	65	20-23*	N/A	1 mm slice thickness	Yes	Yes	No
Liu et al. 2024	N/A	N/A	N/A	N/A	N/A	Yes	No	No
Guo et al. 2025	N/A	N/A	N/A	N/A	N/A	No	Yes	No

*: Range, **: Mean +/- SD, ***: Median, CE-T1: Contrast Enhanced T1-weighted, N/A: Not available

Table S6. Automatic PA Segmentation Study Characteristics : Demographics, Imaging, and Preprocessing Details

**Study**	**Male (%)**	**Female (%)**	**Age**	**Mean PA Volume**	**Imaging Sequence**	**Preprocess**	**Augmentation**	**Post-process**
Diaz-Pernas et al. 2021	N/A	N/A	N/A	N/A	Cheng 2015 data	No	Yes	No
Maas et al. 2021	N/A	N/A	N/A	N/A	Cheng 2015 data	No	No	No
Li et al. 2021	53	47	48.1 +/-11.98**	$11.23cm^3$	T1, T2, FLAIR (Axial, Coronal, Sagittal), CE-T1	Yes	No	No
Wang et al. 2021	55	108	38.64 +/- 12.14**	N/A	N/A	No	No	No
Wu et al. 2021	N/A	N/A	N/A	N/A	CE-T1 Axial	Yes	Yes	No
Shu et al. 2021	51	49	15-76*	N/A	CE-T1 Axial	Yes	Yes	No
Gab Allah et al. 2022	N/A	N/A	N/A	N/A	Cheng 2015 data	Yes	Yes	Yes
Jiang et al. 2022	N/A	N/A	N/A	N/A	N/A	Yes	Yes	No
Razzhagi et al. 2022	N/A	N/A	N/A	N/A	N/A	No	No	No
Li et al. 2023	48	52	5-86*	N/A	T1, T2, CE-T1, CE-T2 Coronal (2–3.5mm)	Yes	No	No
Da Mutten et al. 2023	56	44	55+/-19**	$9.12cm^3$	N/A	Yes	Yes	No
Cerný et al. 2023	50	50	N/A	N/A	CE-T1 Coronal	Yes	Yes	No
Zhang et al. 2023	N/A	N/A	N/A	N/A	N/A	No	No	No
Rai et al. 2024	N/A	N/A	N/A	N/A	Cheng 2015 data	Yes	Yes	Yes
Alqhatani et al. 2024	N/A	N/A	N/A	N/A	Cheng 2015 data	Yes	No	No
Zhang et al. 2024	N/A	N/A	N/A	N/A	N/A	No	Yes	No
Saifullah et al. 2024	N/A	N/A	N/A	N/A	Cheng 2015 data	Yes	No	No
Guo et al. 2024	N/A	N/A	N/A	N/A	N/A	No	Yes	No

*: Range, **: Mean +/- SD, ***: Median, CE-T1: Contrast Enhanced T1-weighted, Cheng 2015 data: CE-T1 Axial, Coronal & Sagittal, FLAIR: Fluid Attenuated Inversion Recovery, N/A: Not available

Table S7. Semi-automated PG Segmentation Study Characteristics : Demographics, Imaging, and Preprocessing Details

Study	Male (%)	Female (%)	Age	Mean PG Volume	Imaging Sequence	Preprocess	Augmentation	Post-process
Zhang et al. 2016	N/A	N/A	N/A	N/A	T1 Axial, Coronal & Sagittal	No	Yes	No
Banday & Mir 2017	N/A	N/A	N/A	N/A	T1, T2, FLAIR, DCE Axial, Coronal & Sagittal (2mm)	Yes	No	No
Zennadi et al. 2024	0	100	20–33* (23+/-3**)	7.05cm^3	3D T1 1mm slice thickness	No	No	No

*: Age range, **: Mean +/- SD, CE-T1: Contrast Enhanced T1-weighted, DCE: Dynamic Contrast Enhanced, FLAIR: Fluid Attenuated Inversion Recovery, N/A: Not available

Table S8. Semi-automated PA Segmentation Study Characteristics : Demographics, Imaging, and Preprocessing Details

Study	Male (%)	Female (%)	Age	Mean PA Volume	Imaging Sequence	Preprocess	Augmentation	Post-process
Zukic et al. 2011	N/A	N/A	N/A	6.3cm^3	N/A	No	No	No
Egger et al. 2012	N/A	N/A	N/A	N/A	T1 Coronal & Axial, T2 Coronal & Axial	No	No	No
Egger et al. 2013	N/A	N/A	N/A	6.3cm^3	T1, T2 Axial, Coronal & Sagittal	No	No	No
Sun et al. 2017	N/A	N/A	N/A	N/A	N/A	No	No	No
Thias et al. 2019	N/A	N/A	N/A	N/A	Cheng 2015 data	No	No	No
Kumar Sa et al. 2020	N/A	N/A	N/A	N/A	Cheng 2015 data	Yes	No	No

Cheng 2015 data: CE-T1 Axial, Coronal & Sagittal, CE-T1: Contrast Enhanced T1-weighted, N/A: Not available

Mixed Method Appraisal Tool (MMAT) Assessment

The MMAT assessment, with a total best score of 100%, was applied to each study based on the following criteria:

0.0.1 1. Appropriateness of Study Design (20%)

- **Description:** Evaluates whether the study design aligns with the research question and objectives.
- **Score:** 0-5 points (0-20%)

0.0.2 2. Sampling (20%)

- **Description:** Assesses the representativeness and appropriateness of the study sample. Studies with broad applicability and diverse ground truths from various databases were awarded higher points.
- **Score:** 0-5 points (0-20%)

0.0.3 3. Data Collection (20%)

- **Description:** Evaluates the reliability and validity of data collection methods, particularly focusing on the reliability of databases used in the studies.
- **Score:** 0-5 points (0-20%)

0.0.4 4. Study's Data Analysis (20%)

- **Description:** Examines the appropriateness and rigor of data analysis methods. Studies incorporating a range of evaluation metrics (e.g., Jaccard, Dice, Hausdorff) received higher consideration.
- **Score:** 0-5 points (0-20%)

0.0.5 5. Researcher's Influence (20%)

- **Description:** Assesses whether the intervention (automatic segmentation) was administered as intended and considers potential biases introduced by the researcher.
- **Score:** 0-5 points (0-20%)

0.0.6 Overall Risk of Bias Score

- **Low Risk of Bias (>70%):** Studies that demonstrated high quality across all criteria.
- **Moderate Risk of Bias(>50% <70%):** Studies with some concerns but overall robust.
- **High Risk of Bias(<50%):** Studies with significant limitations or potential for bias.

Table S9. Risk of Bias Levels of Included Studies

**Risk Level**	**Studies**
Low	[3], [14], [13], [4], [6], [19], [17], [5], [15], [30], [12], [34], [31], [11], [7], [20], [16], [23], [2], [8], [9], [22], [25], [21], [24], [28], [1]
Medium	[33], [29], [27], [32], [10], [18]
High	[26]

Certainty Assessment

The certainty of evidence from the included studies was assessed to ensure that the conclusions drawn were based on reliable and robust data.

0.0.7 Methods Used for Certainty Assessment

The certainty of the evidence was evaluated based on several factors:

1. **Risk of Bias:** The risk of bias within each study was assessed using the Mixed Method Appraisal Tool (MMAT). Studies with lower risk of bias were considered to provide higher certainty evidence.

2. **Reproducibility of Results:** Consideration was given to whether similar results were observed across multiple studies using different cohorts or datasets. Studies that reported consistent outcomes in segmentation performance, particularly in terms of metrics like Dice scores, contributed to higher certainty.

3. **Relevance to the Research Question:** The degree to which the studies directly addressed the research question was considered. Studies that focused specifically on pituitary gland or adenoma segmentation using MRI and reported relevant outcomes were weighted more heavily in the certainty assessment.

4. **Study Design Quality:** Priority was given to studies with rigorous methodological designs, such as those employing well-validated automatic segmentation algorithms, appropriate evaluation metrics, and comprehensive validation approaches.

5. **Data Completeness and Reporting:** The completeness of data reporting, including whether studies provided sufficient details about their methods, results, and potential limitations, was assessed. Studies with more comprehensive and transparent reporting were considered to provide higher certainty evidence.

0.0.8 Overall Certainty of Evidence

Based on the factors outlined above, the certainty of the evidence was categorized as follows:

- **High Certainty:** Studies provided strong, consistent evidence with minimal risk of bias, directly relevant to the research question.
- **Moderate Certainty:** Studies provided reasonably strong evidence, but with some limitations or inconsistencies that might influence the confidence in the results.
- **Low Certainty:** Evidence was provided by studies with significant methodological limitations or inconsistencies, reducing confidence in the results.
- **Very Low Certainty:** Studies provided evidence with substantial limitations, leading to considerable uncertainty in the findings.

Table S10. Certainty of Evidence Across Included Studies

**Certainty Level**	**Studies**
High	[6], [21], [9], [16], [19]
Moderate	[3], [15], [17], [8], [22], [23], [24], [14], [5], [12], [13], [2], [11]
Low	[20], [7], [4], [30], [31], [34], [1], [28], [18], [33], [29], [26], [27], [32], [10]
Very Low	None

Table S11. Summary of Studies Excluded After Full-Text Review

**Reason for Exclusion**	**Study**
No segmentation results for PG or PA provided	[35], [36], [37], [38], [39], [40], [41], [42], [43], [44], [45], [46], [47], [48], [49], [50], [51], [52], [53], [54], [55], [56], [57], [58], [59], [60], [61], [62], [63], [64], [65], [66], [67], [68], [69], [70], [71], [72], [73], [74], [75], [76], [77], [78], [79], [80], [77]
No overlap metrics provided	[81], [82], [83]
Redundant method/Similar approach with a more detailed study already included	[84], [85], [86]
Book chapter	[87], [88]
Segmentation results derived from non-standard Dice and Jaccard formulas	[89]
Retracted Study	[90]

Redundant method/similar approach: studies employing methodologically identical models or pipelines already represented by an included primary study. Non-standard formulas: studies containing invalid or non-standard formulations of segmentation performance metrics.

Table S12. Glossary of Abbreviations Used in the Manuscript

**Abbreviation**	**Full Term**
2D	Two-Dimensional
3D	Three-Dimensional
ABAS	Atlas-Based Automatic Segmentation Software
ABIDE	Autism Brain Imaging Data Exchange
ADNI	Alzheimer's Disease Neuroimaging Initiative
ASSD	Average Symmetric Surface Distance
CALN	Channel Attention (Long-Short-Term-Memory) Network
cfVB-Net	Coarse-to-Fine VB-Net
CNN	Convolutional Neural Network
CONDSEG	Conditional Segmentation
DSC	Dice Similarity Coefficient
FCM	Fuzzy C-Means
Giant PA	Giant Pituitary Adenoma
MACWE	Morphological Active Contour Without Edge
Macro-PA	Macroadenoma
MB	Morphological Based
MGAC	Morphological Geodesic Active Contour
Micro-PA	Microadenoma
MPA	Maximum Probability Atlas
MRI	Magnetic Resonance Imaging
MSR-Net	Multi-Scale Residual Network
N/A	Not Applicable
NFPA	Non-Functioning Pituitary Adenoma
NTUH	National Taiwan University Hospital
OBELISK	Object Boundary Extraction using Learned Image Structures
OVMA	One Voxel Mismatch Allowed
PA	Pituitary Adenoma
PDC U-Net	Parallel Dilated Convolutional U-Net
PG	Pituitary Gland
PSPNet4	Pyramid Scene Parsing Network version 4
Res U-Net	Residual U-Net
SAC	Snake Active Contour
SPM	Statistical Parametric Mapping
U-Net	Convolutional Neural Network architecture for image segmentation
UNET3D	3D version of U-Net
UNETR	U-Net Transformer
VNET	V-Net

References

[1] Aurélie Isambert, Frédéric Dhermain, François Bidault, Olivier Commowick, Pierre-Yves Bondiau, Grégoire Malandain, and Dimitri Lefkopoulos. Evaluation of an atlas-based automatic segmentation software for the delineation of brain organs at risk in a radiation therapy clinical context. *Radiotherapy and Oncology*, 87(1):93–99, 2008.

[2] Pawel Mlynarski, Hervé Delingette, Hamza Alghamdi, Pierre-Yves Bondiau, and Nicholas Ayache. Anatomically consistent cnn-based segmentation of organs-at-risk in cranial radiotherapy. *Journal of Medical Imaging*, 7(1):014502–014502, 2020.

[3] He Wang, Wentai Zhang, Shuo Li, Yanghua Fan, Ming Feng, and Renzhi Wang. Development and evaluation of deep learning-based automated segmentation of pituitary adenoma in clinical task. *The Journal of Clinical Endocrinology & Metabolism*, 106(9):2535–2546, 2021.

[4] Tomoko Maruyama, Norio Hayashi, Yusuke Sato, Toshihiro Ogura, Masumi Uehara, Akio Ogura, Haruyuki Watanabe, Yoshihiro Kitoh, and Alzheimer's Disease Neuroimaging Initiative. Simultaneous brain structure segmentation in magnetic resonance images using deep convolutional neural networks. *Radiological Physics and Technology*, 14:358–365, 2021.

[5] Rachel Gologorsky, Edward Harake, Grace von Oiste, Mustafa Nasir-Moin, William Couldwell, Eric Oermann, and Todd Hollon. Generating novel pituitary datasets from open-source imaging data and deep volumetric segmentation. *Pituitary*, 25(6):842–853, 2022.

[6] Martin Černỳ, Jan Kybic, Martin Májovskỳ, Vojtěch Sedlák, Karin Pirgl, Eva Misiorzová, Radim Lipina, and David Netuka. Fully automated imaging protocol independent system for pituitary adenoma segmentation: a convolutional neural network—based model on sparsely annotated mri. *Neurosurgical Review*, 46(1):116, 2023.

[7] Nouf Alzahrani, Ann Henry, Anna Clark, Louise Murray, Michael Nix, and Bashar Al-Qaisieh. Geometric evaluations of ct and mri based deep learning segmentation for brain oars in radiotherapy. *Physics in Medicine & Biology*, 68(17):175035, 2023.

[8] Uk-Su Choi, Yul-Wan Sung, and Seiji Ogawa. deeppgsegnet: Mri-based pituitary gland segmentation using deep learning. *Frontiers in Endocrinology*, 15:1338743, 2024.

[9] Jingyi Liu, Zhaoze Sun, Qing Guo, Bing Liu, Tianyu Fu, Guolin Ma, Hong Song, and Jian Yang. Caln: Channel attention lstm network for pituitary segmentation in dynamic contrast-enhanced mri. In *2024 IEEE International Symposium on Biomedical Imaging (ISBI)*, pages 1–5. IEEE, 2024.

[10] Te Guo, Jixin Luan, Jingyuan Gao, Bing Liu, Tianyu Shen, Hongwei Yu, Guolin Ma, and Kunfeng Wang. Computer-aided diagnosis of pituitary microadenoma on dynamic contrast-enhanced mri based on spatio-temporal features. *Expert Systems with Applications*, 260:125414, 2025.

[11] Francisco Javier Díaz-Pernas, Mario Martínez-Zarzuela, Míriam Antón-Rodríguez, and David González-Ortega. A deep learning approach for brain tumor classification and segmentation using a multiscale convolutional neural network. *Healthcare*, 9(2):153, 2021.

[12] Benjamin Maas, Erfan Zabeh, and Soroush Arabshahi. Quicktumornet: fast automatic multi-class segmentation of brain tumors. In *2021 10th international IEEE/EMBS conference on neural engineering (NER)*, pages 81–85. IEEE, 2021.

[13] H. Li, Q. Zhao, Y. Zhang, K. Sai, L. Xu, Y. Mou, Y. Xie, J. Ren, and X. Jiang. Image-driven classification of functioning and nonfunctioning pituitary adenoma by deep convolutional neural networks. *Computational and Structural Biotechnology Journal*, 19:3077–3086, 2021.

[14] Siangruei Wu, Yihong Wu, Haoyun Chang, Florence T Su, Hengchun Liao, Wanju Tseng, Chunchih Liao, Feipei Lai, Fengming Hsu, and Furen Xiao. Deep learning-based segmentation of various brain lesions for radiosurgery. *Applied Sciences*, 11(19):9180, 2021.

[15] Xujun Shu, Yijie Zhou, Fangye Li, Tao Zhou, Xianghui Meng, Fuyu Wang, Zhizhong Zhang, Jian Pu, and Bainan Xu. Three-dimensional semantic segmentation of pituitary adenomas based on the deep learning framework-nnu-net: A clinical perspective. *Micromachines*, 12(12):1473, 2021.

[16] Ahmed M Gab Allah, Amany M Sarhan, and Nada M Elshennawy. Edge u-net: Brain tumor segmentation using mri based on deep u-net model with boundary information. *Expert Systems with Applications*, 213:118833, 2023.

[17] Xiaoliang Jiang, Junjian Xiao, Qile Zhang, Lihui Wang, Jinyun Jiang, and Kun Lan. Improved u-net based on cross-layer connection for pituitary adenoma mri image segmentation. *Mathematical biosciences and engineering: MBE*, 20(1):34–51, 2022.

[18] Parvin Razzaghi, Karim Abbasi, Mahmoud Shirazi, and Shima Rashidi. Multimodal brain tumor detection using multimodal deep transfer learning. *Applied Soft Computing*, 129:109631, 2022.

[19] Hongxia Li, Zhiling Liu, Fuyan Li, Feng Shi, Yuwei Xia, Qing Zhou, and Qingshi Zeng. Preoperatively predicting ki67 expression in pituitary adenomas using deep segmentation network and radiomics analysis based on multiparameter mri. *Academic Radiology*, 31(2):617–627, 2023.

[20] Raffaele Da Mutten, Olivier Zanier, Olga Ciobanu-Caraus, Stefanos Voglis, Michael Hugelshofer, Athina Pangalu, Luca Regli, Carlo Serra, and Victor E Staartjes. Automated volumetric assessment of pituitary adenoma. *Endocrine*, 83(1):171–177, 2024.

[21] Qile Zhang, Jianzhen Cheng, Chun Zhou, Xiaoliang Jiang, Yuanxiang Zhang, Jiantao Zeng, and Li Liu. Pdc-net: parallel dilated convolutional network with channel attention mechanism for pituitary adenoma segmentation. *Frontiers in Physiology*, 14:1259877, 2023.

[22] Hari Mohan Rai, Joon Yoo, and Serhii Dashkevych. Two-headed unetefficientnets for parallel execution of segmentation and classification of brain tumors: Incorporating postprocessing techniques with connected component labelling. *Journal of Cancer Research and Clinical Oncology*, 150(4):220, 2024.

[23] Samar M Alqhtani, Toufique A Soomro, Ahmed Ali, Abdul Aziz, Muhammad Irfan, Saifur Rahman, Mohammed Jalalah, Abdulkarem HM Almawgani, and Ladon Ahmed Bade Eljak. Improved brain tumor segmentation and classification in brain mri with fcm-svm: a diagnostic approach. *IEEE Access*, 2024.

[24] Qile Zhang, Xiaoliang Jiang, Xiuqing Huang, and Chun Zhou. Msr-net: Multi-scale residual network based on attention mechanism for pituitary adenoma mri image segmentation. *IEEE Access*, 2024.

[25] Shoffan Saifullah and Rafał Dreżewski. Automatic brain tumor segmentation using convolutional neural networks: U-net framework with pso-tuned hyperparameters. In *International Conference on Parallel Problem Solving from Nature*, pages 333–351. Springer, 2024.

[26] X. Zhang, X. Liu, and W. Lin. Computing optimization technique in enhancing magnetic resonance imaging and brain segmentation of hypophysis cerebri based on morphological based image processing. *Journal of Medical Imaging and Health Informatics*, 6(4):1063–1070, 2016.

[27] Shoaib Amin Banday and Ajaz Hussain Mir. Enhancement and segmentation of pituitary gland from mr brain images. *International Journal of Medical Engineering and Informatics*, 9(3):201–219, 2017.

[28] Manel Merabet Zennadi, Maurice Ptito, Jérôme Redouté, Nicolas Costes, Claire Boutet, Natacha Germain, Bogdan Galusca, and Fabien C Schneider. Mri atlas of the pituitary gland in young female adults. *Brain Structure and Function*, 229(4):1001–1010, 2024.

[29] D. Zukic, J. Egger, M.H. Bauer, D. Kuhnt, B. Carl, B. Freisleben, A. Kolb, and C. Nimsky. Preoperative volume determination for pituitary adenoma. In *Medical Imaging 2011: Computer-Aided Diagnosis*, volume 7963, pages 817–823. SPIE, March 2011.

[30] Jan Egger, Tina Kapur, Christopher Nimsky, and Ron Kikinis. Pituitary adenoma volumetry with 3d slicer. *PLOS ONE*, 7(12):e51788, 2012.

[31] J. Egger, D. Zukić, B. Freisleben, A. Kolb, and C. Nimsky. Segmentation of pituitary adenoma: A graph-based method vs. a balloon inflation method. *Computer Methods and Programs in Biomedicine*, 110(3):268–278, 2013.

[32] M. Sun, X. Chen, Z. Zhang, and C. Ma. Random walk and graph cut based active contour model for three-dimension interactive pituitary adenoma segmentation from mr images. In *Medical Imaging 2017: Image Processing*, volume 10133, pages 548–555. SPIE, February 2017.

[33] A.H. Thias, A.F. Al Mubarok, A. Handayani, D. Danudirdjo, and T.E. Rajab. Brain tumor semi-automatic segmentation on mri t1-weighted images using active contour models. In *2019 International Conference on Mechatronics, Robotics and Systems Engineering (MoRSE)*, pages 217–221. IEEE, December 2019.

[34] Sa Bijay Kumar, Rutuparna Panda, and Sanjay Agrawal. Brain magnetic resonance image tumor detection and segmentation using edgeless active contour. In *2020 11th international conference on computing, communication and networking technologies (ICCCNT)*, pages 1–7. IEEE, 2020.

[35] Sunil Kumar and Dilip Kumar. Human brain tumor classification and segmentation using cnn. *Multimedia Tools and Applications*, 82(5):7599–7620, 2023.

[36] Arshad Hashmi and Ahmed Hamza Osman. Brain tumor classification using conditional segmentation with residual network and attention approach by extreme gradient boost. *Applied Sciences*, 12(21):10791, 2022.

[37] S Divya, L Padma Suresh, and Ansamma John. Enhanced deep-joint segmentation with deep learning networks of glioma tumor for multi-grade classification using mr images. *Pattern Analysis and Applications*, 25(4):891–911, 2022.

[38] OAMF Alnaggar, Basavaraj Ningappa Jagadale, and Swaroopa Hebbar Narayan. Mri brain tumor detection using boosted crossbred random forests and chimp optimization algorithm based convolutional neural networks. *Int J Intell Eng Syst*, 15(2):36–46, 2022.

[39] T. Balamurugan and E. Gnanamanoharan. Genetic algorithm and deep learning feature based tumor detection. *Indian Journal of Computer Science and Engineering*, 12(6):1837–1846, 2021.

[40] Rehna Kalam, Ciza Thomas, and M Abdul Rahiman. Detection of brain tumor in mri images using optimized anfis classifier. *International Journal of Uncertainty, Fuzziness and Knowledge-Based Systems*, 29(Supp01):1–29, 2021.

[41] P Pranav and P Samhita. Automated computer-aided diagnosis for brain tumor detection. In *2021 13th Biomedical Engineering International Conference (BMEiCON)*, pages 1–5. IEEE, 2021.

[42] Sachin Gupta, Narinder Singh Punn, Sanjay Kumar Sonbhadra, and Sonali Agarwal. Mag-net: Multi-task attention guided network for brain tumor segmentation and classification. In *Big Data Analytics: 9th International Conference, BDA 2021, Virtual Event, December 15-18, 2021, Proceedings 9*, pages 3–15. Springer, 2021.

[43] Bala Venkateswarlu Isunuri and Jagadeesh Kakarla. Brain tumor extraction using adaptive threshold selection network. In *2019 IEEE 1st International Conference on Energy, Systems and Information Processing (ICESIP)*, pages 1–6. IEEE, 2019.

[44] Jan Egger, Christoph Kappus, Bernd Freisleben, and Christopher Nimsky. A medical software system for volumetric analysis of cerebral pathologies in magnetic resonance imaging (mri) data. *Journal of medical systems*, 36:2097–2109, 2012.

[45] K Wisaeng and W Sa-Ngiamvibool. Brain tumor segmentation using fuzzy otsu threshold morphological algorithm. *IAENG International Journal of Applied Mathematics*, 53(2):1–12, 2023.

[46] Fathe Jeribi and Uma Perumal. Lesion detection based bt type classification model using svt-kld-fcm and vcr-50. In *International Conference on Computing and Information Technology*, pages 11–25. Springer, 2023.

[47] Sara Ali Abd Al Hussen and Elham Mohammed Thabit A Alsaadi. Automated identification and classification of brain tumors using hybrid machine learning models and mri imaging. *Ingenierie des Systemes d'Information*, 28(5):1299, 2023.

[48] Akshya Kumar Sahoo, Priyadarsan Parida, K Muralibabu, and Sonali Dash. Efficient simultaneous segmentation and classification of brain tumors from mri scans using deep learning. *Biocybernetics and Biomedical Engineering*, 43(3):616–633, 2023.

[49] Nongmeikapam Thoiba Singh, Puneet Kaur, Amrita Chaudhary, and Sanjay Singla. Detection of brain tumors through the application of deep learning and machine learning models. In *2023 IEEE 8th International Conference for Convergence in Technology (I2CT)*, pages 1–6. IEEE, 2023.

[50] Yajuvendra Pratap Singh and DK Lobiyal. A comparative analysis and classification of cancerous brain tumors detection based on classical machine learning and deep transfer learning models. *Multimedia Tools and Applications*, 83(13):39537–39562, 2024.

[51] Narayanan Krishnasamy and Thangaraj Ponnusamy. Deep learning-based robust hybrid approaches for brain tumor classification in magnetic resonance images. *International Journal of Imaging Systems and Technology*, 33(6):2157–2177, 2023.

[52] Patrizia Vizza, Mattia Cannistrà, Raffaele Giancotti, and Pierangelo Veltri. Image processing segmentation algorithms evaluation through implementation choices. In *Proceedings of the 13th ACM International Conference on Bioinformatics, Computational Biology and Health Informatics*, pages 1–7, 2022.

[53] Jyotismita Chaki and Marcin Woźniak. A deep learning based four-fold approach to classify brain mri: Btscnet. *Biomedical Signal Processing and Control*, 85:104902, 2023.

[54] Alwas Muis, Sunardi Sunardi, and Anton Yudhana. Comparison analysis of brain image classification based on thresholding segmentation with convolutional neural network. *Journal of Applied Engineering and Technological Science (JAETS)*, 4(2):664–673, 2023.

[55] Gurjot Singh and Vinay Gautam. Pituitary gland size estimation and lesion detection using segmentation-based color thresholding technique. In *2023 International Conference on Sustainable Computing and Smart Systems (ICSCSS)*, pages 700–704. IEEE, 2023.

[56] Ayan Gupta, Mayank Dixit, Vipul Kumar Mishra, Attulya Singh, and Atul Dayal. Brain tumor segmentation from mri images using deep learning techniques. In *International Advanced Computing Conference*, pages 434–448. Springer, 2023.

[57] Sara Ali Abd Al Hussen and Elham Mohammed Thabit A Alsaadi. Automated identification and classification of brain tumors using hybrid machine learning models and mri imaging. *Ingenierie des Systemes d'Information*, 28(5):1299, 2023.

[58] Shirin Kordnoori, Maliheh Sabeti, Mohammad Hossein Shakoor, and Ehsan Moradi. Deep multi-task learning structure for segmentation and classification of supratentorial brain tumors in mr images. *Interdisciplinary Neurosurgery*, 36:101931, 2024.

[59] Sumaiya Deen Muhammad and Ziad Kobti. An ensemble deep learning approach for enhanced classification of pituitary tumors. In *2023 IEEE Symposium Series on Computational Intelligence (SSCI)*, pages 427–432. IEEE, 2023.

[60] Jayasri Kotti, Manikandan Moovendran, and Mekala Kandasamy. Multi-level brain tumor classification using hybrid coot flamingo search optimization algorithm enabled deep learning with mri images. *Network: Computation in Neural Systems*, pages 1–32, 2024.

[61] K Shanthala, BM Chandrakala, N Shobha, et al. Automated diagnosis of brain tumor classification and segmentation of mri images. In *2023 International Conference on the Confluence of Advancements in Robotics, Vision and Interdisciplinary Technology Management (IC-RVITM)*, pages 1–7. IEEE, 2023.

[62] Pavihaa Lakshmi Babu Muthu Krishnan and Vidhya Sampath. A multiscale morphological segmentation and classification of brain tumor using supervised learning algorithm. In *AIP Conference Proceedings*, volume 2966. AIP Publishing, 2024.

[63] Bhumika Mehta, Richa Sharma, Sunil Kumar, Sushil Singh Chauhan, and Vipin Bhatnagar. Computer vision tools for tumor segmentation. In *High Energy Physics Symposium*, pages 550–554. Springer, 2022.

[64] Lehel Dénes-Fazekas, Levente Kovács, György Eigner, and László Szilágyi. Brain tumor segmentation from multi-spectral mri records using a u-net cascade architecture. In *2023 IEEE International Conference on Systems, Man, and Cybernetics (SMC)*, pages 3003–3008. IEEE, 2023.

[65] Ouiza Nait Belaid, Malik Loudini, and Amir Nakib. Brain tumor classification using densenet and u-net convolutional neural networks. In *2024 8th International Conference on Image and Signal Processing and their Applications (ISPA)*, pages 1–6. IEEE, 2024.

[66] Sukanya Roy, Bijoy Kumar Mandal, Koustov Khamaru, and Arghya Bhattacharya. Prediction and classification of brain tumor using modified transfer learning. In *2024 11th International Conference on Computing for Sustainable Global Development (INDIACom)*, pages 850–854. IEEE, 2024.

[67] Yogesh Kumar, Veena S Badiger, Gopal K Shyam, et al. Brain tumour segmentation and classification using the convolutional neural network (u-net model). In *2023 International Conference on Advanced Computing & Communication Technologies (ICACCTech)*, pages 258–265. IEEE, 2023.

[68] Sofia Rosa, Verónica Vasconcelos, and Pedro JSB Caridade. Evaluating the impact of filtering techniques on deep learning-based brain tumour segmentation. *Computers*, 13(9):237, 2024.

[69] Parasa Rishi Kumar, Kavya Bonthu, Boyapati Meghana, Koneru Suvarna Vani, and Prasun Chakrabarti. Multi-class brain tumor classification and segmentation using hybrid deep learning network model. *Scalable Computing: Practice and Experience*, 24(1):69–80, 2023.

[70] M Geetha, V Srinadh, J Janet, and S Sumathi. Hybrid archimedes sine cosine optimization enabled deep learning for multilevel brain tumor classification using mri images. *Biomedical Signal Processing and Control*, 87:105419, 2024.

[71] Sandhya Sandeep Waghere and Jayashri Prashant Shinde. A robust classification of brain tumor disease in mri using twin-attention based dense convolutional auto-encoder. *Biomedical Signal Processing and Control*, 92:106088, 2024.

[72] Gaoxiang Li, Xiao Hui, Wenjing Li, and Yanlin Luo. Multitask learning with multiscale residual attention for brain tumor segmentation and classification. *Machine Intelligence Research*, 20(6):897–908, 2023.

[73] Faisal Alshomrani. A unified pipeline for simultaneous brain tumor classification and segmentation using fine-tuned cnn and residual unet architecture. *Life*, 14(9):1143, 2024.

[74] Ifrah Andleeb, B Zahid Hussain, Salik Ansari, Mohammad Samar Ansari, Nadia Kanwal, and Asra Aslam. Deep learning based lightweight model for brain tumor classification and segmentation. In *UK Workshop on Computational Intelligence*, pages 491–503. Springer, 2023.

[75] Rasool Fakhir Jader, Shahab Wahhab Kareem, and Hoshang Qasim Awla. Ensemble deep learning technique for detecting mri brain tumor. *Applied Computational Intelligence and Soft Computing*, 2024(1):6615468, 2024.

[76] Rudresh Deepak Shirwaikar, Kruthika Ramesh, Abu Mohammed Faisal, M Jeshwanth, and Aditya Raghav. 3d segmentation of brain tumour. *International Journal of Engineering Systems Modelling and Simulation*, 15(2):76–83, 2024.

[77] S Sindhu and N Vijayalakshmi. The elevation of efficacy identifying pituitary tissue abnormalities within brain images by employing memory contrast learning techniques. *Journal of applied mathematics & informatics*, 42(4):931–943, 2024.

[78] Xiaorong Yan, Bingquan Lin, Jun Fu, Shuo Li, He Wang, Wenjian Fan, Yanghua Fan, Ming Feng, Renzhi Wang, Jun Fan, et al. Deep-learning-based automatic segmentation and classification for craniopharyngiomas. *Frontiers in Oncology*, 13:1048841, 2023.

[79] Sagrika Gargya and Shruti Jain. Cad system design for pituitary tumor classification based on transfer learning technique. *Current Medical Imaging*, 20(1):E15734056246146, 2024.

[80] Maram Fahaad Almufareh, Muhammad Imran, Abdullah Khan, Mamoona Humayun, and Muhammad Asim. Automated brain tumor segmentation and classification in mri using yolo-based deep learning. *IEEE Access*, 12:16189–16207, 2024.

[81] Diane M Renz, Horst K Hahn, Peter Schmidt, Jan Rexilius, Markus Lentschig, Alexander Pfeil, Dieter Sauner, Clemens Fitzek, Hans-Joachim Mentzel, Werner A Kaiser, et al. Accuracy and reproducibility of a novel semi-automatic segmentation technique for mr volumetry of the pituitary gland. *Neuroradiology*, 53:233–244, 2011.

[82] Angelita Pui-Yee Wong, Jon Pipitone, Min Tae M Park, Erin W Dickie, Gabriel Leonard, Michel Perron, Bruce G Pike, Louis Richer, Suzanne Veillette, M Mallar Chakravarty, et al. Estimating volumes of the pituitary gland from t1-weighted magnetic-resonance images: effects of age, puberty, testosterone, and estradiol. *Neuroimage*, 94:216–221, 2014.

[83] Qasim Al Hinai, Kelvin Mok, Anthony Zeitouni, Bruno Gagnon, Abdul Razag Ajlan, Juan Rivera, Marc Tewfik, and Denis Sirhan. Comparison between manual and semiautomated volumetric measurements of pituitary adenomas. *Skull Base*, 21(06):365–372, 2011.

[84] Samar M Alqhtani, Toufique Ahmed Soomro, Ahmed Ali, Muhammad Irfan, Abdullah A Asiri, et al. Contrast normalization strategies in brain tumor imaging: From preprocessing to classification. *CMES Computer Modeling in Engineering and Sciences*, 140(2), 2024.

[85] Abdullah A Asiri, Toufique Ahmed Soomro, Ahmed Ali Shah, Ganna Pogrebna, Muhammad Irfan, and Saeed Alqahtani. Optimized brain tumor detection: a dual-module approach for mri image enhancement and tumor classification. *IEEE Access*, 12:42868–42887, 2024.

[86] Hongxia Li, Zhiling Liu, Fuyan Li, Yuwei Xia, Tong Zhang, Feng Shi, and Qingshi Zeng. Identification of prolactinoma in pituitary neuroendocrine tumors using radiomics analysis based on multiparameter mri. *Journal of Imaging Informatics in Medicine*, pages 1–9, 2024.

[87] V Abinash, S Meghanth, P Rakesh, SA Sajidha, VM Nisha, and A Muralidhar. An efficient transfer learning–based cnn multi-label classification and resunet based segmentation of brain tumor in mri. In *Recurrent Neural Networks*, pages 247–262. CRC Press, Boca Raton, FL, 2022.

[88] R Rajagopal and S Edwin Jose. An efficient framework for locating stroke in brain mri images using radon transform and convolutional neural networks. In *Next Generation of Internet of Things: Proceedings of ICNGIoT 2021*, pages 385–395. Springer, 2021.

[89] GS Sunsuhi and S Albin Jose. An adaptive eroded deep convolutional neural network for brain image segmentation and classification using inception resnetv2. *Biomedical Signal Processing and Control*, 78:103863, 2022.

[90] Ejaz Ul Haq, Huang Jianjun, Xu Huarong, Kang Li, and Lifen Weng. [retracted] a hybrid approach based on deep cnn and machine learning classifiers for the tumor segmentation and classification in brain mri. *Computational and Mathematical Methods in Medicine*, 2022(1):6446680, 2022.
